# Supplementary material for: Strategies to identify and suppress crosstalk signals in double electron–electron resonance (DEER) experiments with gadoliniumIII and nitroxide spin-labeled compounds
Source: Magn Reson (Gott). 2020 Dec 9;1(2):285–99. doi: 10.5194/mr-1-285-2020 (PMC10500692; doi:10.5194/mr-1-285-2020)
Supplement: The supplement related to this article is available online at: https://doi.org/10.5194/mr-1-285-2020-supplement. [file mr-1-285-supplement.pdf]

Supplement of Magn. Reson., 1, 285–299, 2020
https://doi.org/10.5194/mr-1-285-2020-supplement
© Author(s) 2020. This work is distributed under
the Creative Commons Attribution 4.0 License.

Open Access
MAGNETIC RESONANCE

Supplement of

Strategies to identify and suppress crosstalk signals in double electron–electron resonance (DEER) experiments with gadolinium^{III} and nitroxide spin-labeled compounds

Markus Teucher et al.

Correspondence to: Enrica Bordignon (enrica.bordignon@rub.de)

The copyright of individual parts of the supplement might differ from the CC BY 4.0 License.

SI part A

Details of the syntheses

Contents

Syntheses of NO-NO ruler **12** **2**
Experimental part **4**
References **11**
NMR spectra **12**

Syntheses of NO-NO ruler 12

For this study, a water-soluble NO-NO ruler with a short and well-defined spin-spin distance was needed. In the past we had worked with NO-NO rulers fulfilling the requirement on the distance, but being insoluble in water.[1] To achieve water solubility, oligo(ethyleneglycol) (PEG) substituents were implemented as side chains. The same approach had been used for obtaining water soluble Gd-Gd and Gd-NO rulers.[2,3]

The synthetic route to NO-NO ruler **12** is depicted in Scheme S1. This ruler was assembled through Pd/Cu-catalyzed alkynyl aryl coupling of pegylated diiodobenzene **11**, a known building block,[2] with alkyne **10** contributing the nitroxide moiety. Both building blocks carry PEG substituents. Alkyne **10** was synthesized starting from acetaminophenol **1**. Firstly, acetaminophenol **1** was iodinated and the product furnished with PEG substituents. Unexpectedly, partial amide hydrolysis occurred during pegylation giving a mixture of amide **4** and amine **5a**. This hydrolysis is not of concern when amine **5a** is the next synthetic target. However, we had to separate the two products for their unambiguous identification. Isolated amide **4** was then converted into amine **5a**. Considering the partial amide hydrolysis during pegylation, we were surprised to find that heating to 85 ^{o}C for more than 5 h was needed for amide hydrolysis through treatment with NaOH in aqueous methanol. The prolonged heating caused deiodination to a small extent (6%). The deiodinated amine **5b** was not removed. It did not interfere with the next synthesis step and was removed thereafter. Having amine **5a** in hand, the alkyne unit, which is relevant for the final ruler assembly, was installed via Pd/Cu-catalyzed alkynyl aryl coupling with (triisopropylsilyl)acetylene and the nitroxide moiety was attached through amide formation. Finally the triisopropyl group was removed to obtain the building block alkyne **10**.

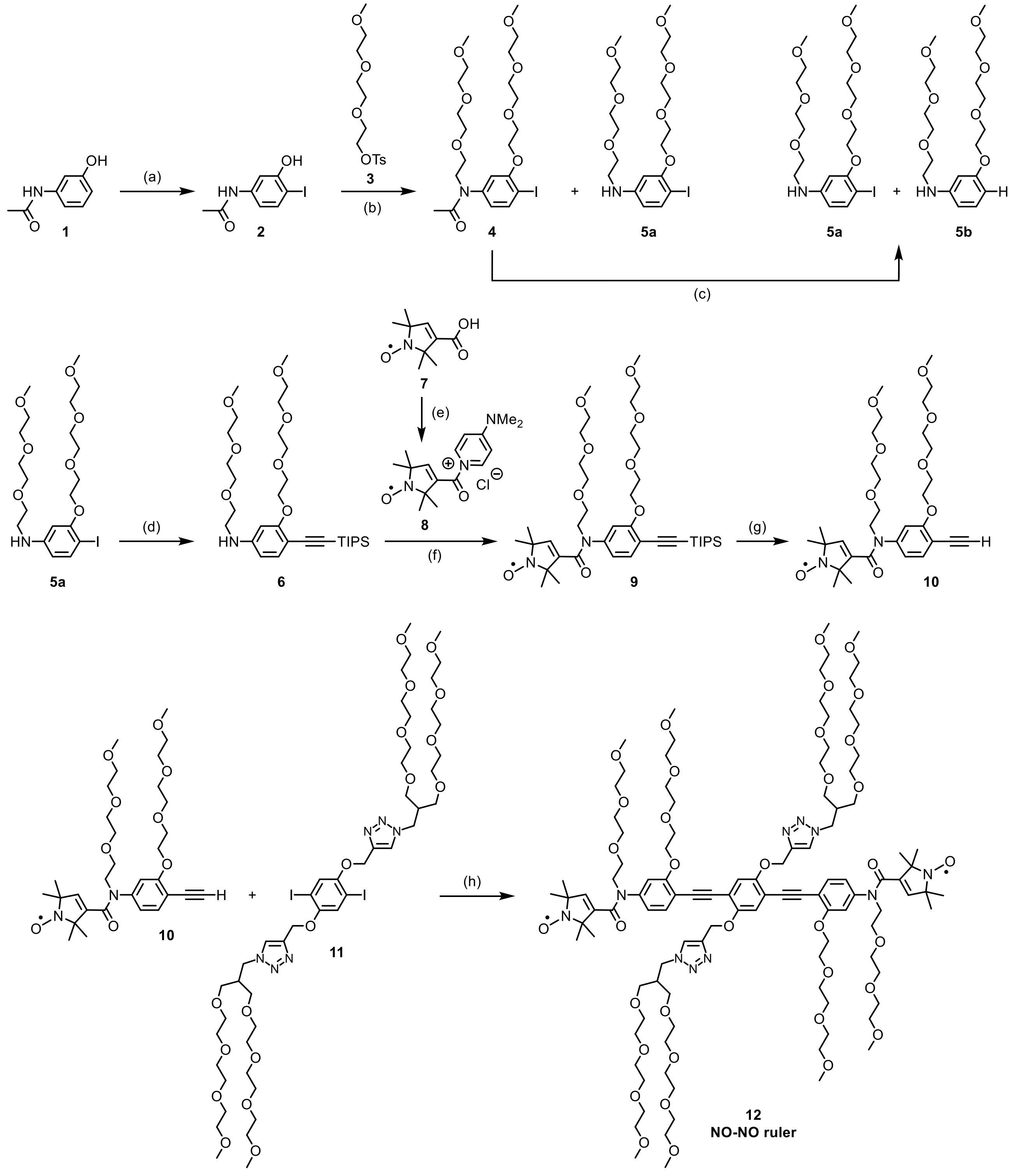

Scheme S1: Synthesis of building blocks and assembly of NO-NO ruler **12**. (a) KI, KIO_3, H_2SO_4, H_2O, rt, 3 h, 84%; (b) NaH, THF, 60 °C, 5 d, yield of amide **4**: 59%, yield of amine **5a**: 17%; (c) NaOH, MeOH, H_2O, 85 °C, 46 h, yield of amine **5a**: 81%, yield of amine **5b**: 6%; (d) HC≡C-TIPS, piperidine, THF, CuI, $Pd(PPh_3)_2Cl_2$, rt, 4 d, 76%; (e) (1) DMAP, CH_2Cl_2, (2) thionyl chloride, 0 °C to rt, 3 h; nitroxide **8** was not isolated; (f) rt, 1 d, 95%; (g) Bu_4NF, THF; rt, 30 min, 100%; (h) THF, piperidine, CuI, $Pd(PPh_3)_2Cl_2$, 45 °C, 3 d, 35%. TIPS = triisopropylsilyl, DMAP = 4-*N*,*N*-dimethylaminopyridine, THF = tetrahydrofuran, rt = room temperature.

Experimental part

General

Unless otherwise stated, reactions were performed under ambient conditions using commercial solvents and reagents. The solvents used for extraction and chromatography were of technical grade and were distilled prior to their use. THF (HPLC grade) was dried with sodium/benzophenone.

The temperatures given for the reactions refer to the bath temperatures. Solvents were removed at a bath temperature of ca. 40 ^{o}C and reduced pressure. The products were dried at room temperature at ca. 0.05 mbar. The pH values of the solutions were determined using pH indicator strips (resolution: 0.3 pH, Merck).

Column chromatography was carried out on silica gel 60 M (Macherey Nagel) applying slight pressure. In the procedures reported below the size of the column is given as diameter × length. The material was loaded onto the column dissolved in a small quantity of the eluent. Thin layer chromatography (TLC) was performed on silica gel coated aluminum foil (Merck, 60 F254). The spots were detected with UV light of λ = 254 and 366 nm. The compositions of solvent mixtures are given in volume ratios.

NMR spectra were calibrated using the solvent signal as an internal standard [$CDCl_3$: $\delta(^1H)$ = 7.26, $\delta(^{13}C\{^1H\})$ = 77.16; CD_2Cl_2: $\delta(^1H)$ = 5.32, $\delta(^{13}C\{^1H\})$ = 54.00; DMSO-d_6: $\delta(^1H)$ = 2.50, $\delta(^{13}C\{^1H\})$ = 39.52]. Signal assignments are supported by DEPT-135, COSY, HMBC and HMQC experiments.

EI mass spectra were recorded using an Autospec X magnetic sector mass spectrometer with EBE geometry (Vacuum Generators, Manchester, UK) equipped with a standard EI source. ESI mass spectra were recorded using an Esquire 3000 ion trap mass spectrometer (Bruker Daltonik GmbH, Bremen, Germany) equipped with a nano-ESI source. ESI accurate mass measurements were acquired using an Agilent 6220 time-of-flight mass spectrometer

(Agilent Technologies, Santa Clara, CA, USA) in extended dynamic range mode equipped with a Dual-ESI source or using a Q-IMS-TOF mass spectrometer Synapt G2Si (Waters GmbH, Manchester, UK) in resolution mode interfaced to a nano-ESI ion source.

The ratio of the components in a mixture was determined by ^{1}H NMR spectroscopy and is given in a molar ratio.

Syntheses

2-Iodo-5-acetaminophenol (2). The published procedure[4] was followed making small changes. A solution of KI (9.7 g, 58.6 mmol) in H_2O (250 mL) and a solution of KIO_3 (6.1 g, 28.6 mmol) in H_2SO_4 (95%, 9.0 g) and H_2O (250 mL) were separately but simultaneously added to a solution of 3-acetaminophenol (**1**) (13.0 g, 86.0 mmol) in aqueous H_2SO_4 (0.05 mol·L^{-1}, 1.5 L) within 2.5 h at such a rate that the addition rate of the solution of KIO_3 was slightly faster than the addition rate of the solution of KI. Pale yellow crystals precipitated during the addition. The suspension was stirred at room temperature for 1.5 h. The precipitate was collected by filtration, dried in a desiccator over P_4O_{10} at reduced pressure for 2 d and recrystallized in acetone. 2-Iodo-5-acetaminophenol (**2**) (20.0 g, 84%) was obtained as colorless crystals. ^{1}H NMR (500 MHz, DMSO-d$_6$): δ = 10.28 (br s, 1 H, NH), 9.91 (s, 1 H, OH), 7.51 (d, ^3J = 8.5 Hz, 1 H, H *ortho* to I), 7.43 (d, ^4J = 2.3 Hz, 1 H, H *ortho* to OH), 6.75 (dd, ^3J = 8.5 Hz, ^4J = 2.3 Hz, 1 H, H *para* to OH), 2.01 (s, 3 H, CH_3). ^{13}C NMR (126 MHz, $CDCl_3$): δ = 168.4 (C=O), 156.6 (C_{Ar}O), 140.6 (C_{Ar}N), 138.3 (CH *ortho* to I), 112.0 (CH *para* to OH), 105.8 (CH *ortho* to OH), 76.4 (C_{Ar}I), 24.1 (CH_3). MS (EI, 70 eV) *m/z* (%) = 277.0 (66) $[M]^{+\bullet}$, 234.9 (100) $[M - Ac]^+$, 108.0 (9), 80.0 (21).

PEG-tosylate 3. Our procedure deviates slightly from the published one.[5] Under cooling with an ice-water-bath, tosylchloride (14.45 g, 75.8 mmol) was added to a solution of $CH_3(OCH_2CH_2)_3OH$ (10.8 g, 65.8 mmol) in pyridine (10.8 mL, 131 mmol). The pale-yellow

solution was stirred at 0 °C for 3.5 h and afterwards aqueous HCl (10%, 120 mL) was added. The solution was extracted with toluene (3 x 75 mL), dried over $MgSO_4$ and the solvent was removed under reduced pressure. Column chromatography (6.5 cm x 43 cm CH_2Cl_2/Et_2O, 1:1) of the residual colorless oil (21.2 g) gave two fractions. The first fraction contained only PEG-tosylate **3** (0.53 g; R_f = 0.40). The second fraction (18.0 g; R_f = 0.40 and 0.42) was a mixture of PEG-tosylate **3** (17.68 g) and tosyl chloride (0.32 g). The tosyl chloride in this fraction was removed easily applying the reported procedure:[6] The second fraction was dissolved in CH_2Cl_2 (80 mL) and pyridine (2.80 mL, 34.5 mmol). Chopped filter paper (2.80 g) was added to the solution and the suspension was treated in an ultrasound-bath for 1 h. Then the suspension was filtered through filter paper, the filter cake was washed with CH_2Cl_2 (100 mL) and the combined filtrates were washed with aqueous HCl (1 M, 50 mL). The layers were separated and the aqueous layer was extracted with CH_2Cl_2 (25 mL). The combined organic layers were dried over magnesium sulfate. After removal of the solvents the residue was combined with the above mentioned first chromatographic fraction. PEG-tosylate **3** (18.2 g, 83 %) was obtained as a colourless oil. ^{1}H NMR (500 MHz, $CDCl_3$): δ [ppm] = 7.80 (AA'-part of a AA'XX' spin system, 2H, H *ortho* to SO_3), 7.34 (XX'-part of the AA'XX' spin system, 2 H, H *ortho* to Me), 4.16 (m, 2 H, TsO-C$\underline{H}_2$CH$_2$), 3.69 (m, 2 H, TsO-CH$_2$C$\underline{H}_2$), 3.60 (m, 6 H, TsO-CH$_2$CH$_2$O-C$\underline{H}_2$C$\underline{H}_2$O-C$\underline{H}_2$CH$_2$OCH$_3$), 3.53 (m, 2 H, TsO-(CH$_2$CH$_2$O)$_2$-CH$_2$C$\underline{H}_2$O-CH$_3$), 3.37 (s, 3 H, OCH$_3$), 2.45 (s, 3 H, ArC$\underline{H}_3$). The shifts of the aromatic protons is significantly different from those reported for this compound.[5] However, the shifts that we determined fit well to the shifts reported in the same reference for PEG-tosylates with other lengths of the PEG chain.

Pegylated amide 4. This reaction was performed in dried glassware under argon using the Schlenk technique. NaH (60 wt% dispersion in mineral oil, 735 mg, 18.4 mmol) was suspended in dry THF (20 mL). 2-Iod-5-acetaminophenol (**2**) (2.00 g, 7.23 mmol) was added slowly in portions (caution: strong gas development and foaming). The milky viscous

suspension was stirred at room temperature for 5 min. PEG-tosylate **3** (4.96 g, 15.57 mmol) was added, whereupon the color of the suspension turned from white to slight yellow. Dry THF (10 mL) was added to reduce the viscosity of the suspension, whereupon the suspension took on a beige color. The suspension was stirred at 60 °C for 70 h. Under cooling with an ice-water bath, aqueous HCl (1 M, 1 mL) and CH_2Cl_2 (20 mL) were added. Additional aqueous HCl (1 M, 2.5 mL) was added to dissolve the precipitate. Water (15 mL) was added, the organic phase was separated, and the aqueous phase was extracted with CH_2Cl_2 (3 x 20 mL). The combined organic phases were washed with saturated aqueous Na_2CO_3 solution (20 mL) and then with water (20 mL), dried over Na_2SO_4, and filtered. The solvents of the filtrate were removed. Chromatography (5 cm x 52 cm, CH_2Cl_2/EtOH, 20:1) of the residual brown oil gave pegylated amide **4** (2.42 g, 59%; R_f = 0.30) as a pale yellow oil and pegylated amine **5a** (0.65 g, 17%; R_f = 0.42) as a green oil.

Analytical data of pegylated amide **4**: ^{1}H NMR (500 MHz, $CDCl_3$) δ [ppm] = 7.77 (d, ^3J = 8.2 Hz, 1H, H *ortho* to I), 6.74 (d, ^4J = 1.5 Hz, 1H, H *ortho* to O), 6.64 (dd, ^3J = 8.2 Hz, ^4J = 1.5 Hz, 1H, H *para* to O), 4.15 and 3.93 (2 m, 2 H each, CH_2), 3.82 and 3.69 (2 m, 4 H each, CH_2 of PEG), 3.59 (m, 10 H, CH_2 of PEG), 3.52 (m, 2 H, CH_2 of PEG), 3.38 and 3.36 (2 s, 3 H each, OCH_3), 1.86 (s, 3 H, C(=O)CH_3). ^{13}C NMR (126 MHz, $CDCl_3$) δ [ppm] = 170.2 (C=O), 158.3 (C_{Ar}O), 144.8 (C_{Ar}N), 139.8 (CH *ortho* to I), 122.4 (CH *para* to O), 112.4 (CH *ortho* to O), 85.5 (C_{Ar}-I), 71.88, 71.86, 71.2, 70.7, 70.5, 70.43, 70.41, 70.0, 69.4, 69.3 and 68.1 (CH_2 of PEG-chain), 58.94 and 58.05 (OCH_3), 22.7 (C(=O)$\underline{C}H_3$). MS (ESI): m/z = 592.2 $[M + Na]^+$.

Analytical data of pegylated amine **5a**: ^{1}H NMR (500 MHz, $CDCl_3$) δ [ppm] = 7.44 (d, ^3J = 8.5 Hz, 1 H, H *ortho* to I), 6.16 (d, ^4J = 2.3 Hz, 1 H, H *ortho* to O), 6.05 (dd, ^3J = 8.5 Hz, ^4J = 2.3 Hz, 1 H, H *para* to O), 4.25 (br s, 1 H, NH), 4.11, 3.90 und 3.82 (3 m, 2 H each, CH_2 of

PEG), 3.66 (m, 12 H, CH_2 of PEG), 3.55 (m, 4 H, CH_2 of PEG), 3.381 and 3.376 (2 s, 3 H each, OCH_3), 3.25 (m, 2 H, NCH_2). MS (ESI): *m/z* = 550.2 $[M + Na]^+$.

Pegylated amine 5a. Pegylated amide **4** (2.42 g, 4.26 mmol) was dissolved in methanol (15 mL), water (15 mL) and aqueous NaOH solution (2 M, 10 mL). The solution was stirred at 85 °C for 5 h. TLC showed an incomplete conversion. NaOH (0.669 g, 16.73 mmol) and methanol (8 mL) were added. The solution was stirred at 85 °C for 41 h. The brown solution was cooled to room temperature and extracted with CH_2Cl_2 (3 x 15 mL), the organic phase was dried over Na_2SO_4, and the suspension was filtered. The solvents of the filtrate were removed. Chromatography (3.5 cm x 53 cm, CH_2Cl_2/EtOH, 20:1) of the residual orange-brown oil (2.08 g) gave a mixture (1.88 g) of pegylated amine **5a** (1.80 g, yield: 81%) and the corresponding deiodinated amine **5b** (83 mg, yield: 6%) as a green oil. This material was used for the next reaction without further treatment. Analytical data of amine **5a** are given above.

TIPS-protected alkyne 6. This reaction was performed under argon using the Schlenk technique. Pegylated amine **5a** (194 mg, 367 μmol), piperidine (0.38 mL, 3.84 mmol), and TIPS-acetylene (123 μL, 550 μmol) was dissolved in THF (6 mL). The solution was degassed through three freeze-pump-thaw cycles. CuI (4.41 mg, 21.2 μmol) and $Pd(PPh_3)_2Cl_2$ (2.13 mg, 3.04 μmol) were added and the pale yellow reaction solution was stirred at room temperature for 96 h, during which a precipitate formed. Water (5 mL) and CH_2Cl_2 (10 mL) were added. Water (10 mL) was added and the phases were separated. The aqueous phase was extracted with CH_2Cl_2 (3 x 10 mL). The combined organic phases were washed with water (2 x 10 mL), dried over Na_2SO_4 and filtered. The solvents were removed. Chromatography (3 cm x 17 cm, CH_2Cl_2/EtOH, 15:1, a few drops of Et_3N were added to the eluent at the beginning of the elution) of the residual yellow oil gave TIPS-protected alkyne **6** (163 mg, 76%; R_f = 0.41) as a brown oil. ^{1}H NMR (500 MHz, $CDCl_3$) δ

[ppm] = 7.22 (d, ^3J = 8.3 Hz, 1 H, H *ortho* to C≡CTIPS), 6.14 (dd, ^3J = 8.6 Hz, ^4J = 1.7 Hz, 1 H, H *para* to O), 6.09 (d, ^4J = 1.5 Hz, 1 H, H *ortho* to O), 4.34 (s, 1 H, NH), 4.13, 3.85, 3.75, and 3.69 (4 m, 2 H each, CH_2 of PEG), 3.66 (m, 10 H, CH_2 of PEG), 3.56 (m, 4 H, CH_2 of PEG), 3.38 and 3.37 (2 s, 3 H each, OCH_3), 3.28 (m, 2 H, CH_2N), 1.11 (s, 21 H, $CH(CH_3)_2$).

Nitroxide 9. This reaction was performed in dried glassware under argon using the Schlenk technique. Under cooling with an ice-water bath, thionyl chloride (40.8 µL, 561 µmol) was added to a yellow solution of 2,2,5,5-tetramethyl-3-pyrrolin-1-oxyl-3-carboxylic acid (**7**) (112 mg, 606 µmol) and DMAP (178 mg, 1.46 mmol) in dry CH_2Cl_2 (7 mL), which made the color of the solution to change from yellow to red. The ice-water bath was removed and the red solution was stirred at room temperature for 3 h. A solution of TIPS-protected alkyne **6** (149.5 mg, 0.257 mmol) in CH_2Cl_2 (3 mL) was added. The solution was stirred at room temperature for 1 d. During this time the color of the solution changed to yellow. The solution was filtered through silica gel (1.7 cm x 2 cm, rinsing with CH_2Cl_2/EtOH, 15:1). The filtrate was washed with aqueous HCl (1 M, 8 mL), saturated aqueous Na_2CO_3 solution (10 mL), and water (10 mL), dried over Na_2SO_4, and filtered. The solvents of the filtrate were removed. Chromatography (3 cm x 10 cm, CH_2Cl_2/EtOH 20:1) of the residue gave nitroxide **9** (183 mg, 95%; R_f = 0.22) as a pale-yellow oil. ^{1}H NMR (500 MHz, CD_2Cl_2): All signals are broad and featureless and integrals in the low field region come with a large error. δ [ppm] = 7.43 (very br s, 1 H, H *ortho* to C≡C-TIPS), 6.85 (very br s, 1.7 H, both H *meta* to C≡C-TIPS), 4.18 (br s, 3.6 H, 2 CH_2 of PEG), 3.88 (br s, 2 H, CH_2 of PEG), 3.71 - 3.53 (m, 18 H, CH_2 of PEG), 3.37 (br s, 6 H, OCH_3), 1.17 (s, 21 H, $C\underline{H}(C\underline{H}_3)_2$). ^{13}C NMR (126 MHz, CD_2Cl_2) δ [ppm] = 159.1 ($C_{Ar}O$), 143.8 (br, $C_{Ar}N$), 132.4 ($C_{Ar}H$ *ortho* to C≡C-TIPS), 119.7 ($C_{Ar}H$ *para* to O), 111.3 and 110.8 ($C_{Ar}H$ *ortho* to O and $\underline{C}_{Ar}$-C≡C-TIPS), 100.8 ($\underline{C}$≡C-TIPS), 94.7 (C≡$\underline{C}$-TIPS), 71.12, 71.06, 69.94, 69.62, 69.60, 69.48, 69.45, 69.23, 68.49, 68.09, and 67.55 (CH_2 of PEG), 57.82 and 57.78 (OCH_3), 46.7 (NCH_2), 17.4 ($CH(\underline{C}H_3)_2$), 10.2 ($\underline{C}H(CH_3)_2$). MS (ESI)

m/z = 770.6 [M + Na]$^+$, 748.6 [M + H]$^+$. Accurate MS (ESI) calcd. for [M + Na]$^+$ $C_{40}H_{67}N_2O_9SiNa^+$: 770.4508; found: 770.4498.

Alkyne 10. A solution of Bu_4NF in THF (1 M, 0.3 mL, 0.3 mmol) was added to a solution of nitroxide **9** (185.6 mg, 0.248 mmol) in THF (6 mL). The solution was stirred at room temperature for 30 min. The solution was filtered through silica gel (1.7 cm x 2 cm, rinsing with CH_2Cl_2/EtOH, 15:1). After removal of the solvents, a mixture (167 mg) of alkyne **10** and TIPS-X (X most probably being F) and Bu_4NY (Y = F, OH) was obtained. This mixture was used for the next reaction without further purification. ^{1}H NMR (500 MHz, CD_2Cl_2): All signals are broad and featureless and integrals in the low field region come with a large error. δ [ppm] = 7.43 (br s, 0.9 H, H *ortho* to C≡C-H), 6.83 (br s, 1.8 H, both H *meta* to C≡C-H), 4.14 (br s, 3.6 H, 2 CH_2 of PEG), 3.9 (br s, 2 H, CH_2 of PEG), 3.72 - 3.50 (m, 16 H, CH_2 of PEG), 3.34 and 3.32 (2 br s, 5.5 H, 2 OCH_3 and C≡C-H), 3.26 (m, 0.8 H, NCH_2).

NO-NO ruler 12. This reaction was performed under argon using the Schlenk technique. The material obtained through desilylation of nitroxide **9** (109.4 mg, ca. 0.185 mmol of alkyne **10**) and diiodo benzene **11**[2] (101.2 mg, 78.79 µmol) were dissolved in piperidine (90 µL, 0.9 mmol) and THF (6 mL). The solution was degassed through three freeze-pump-thaw cycles. CuI (1.29 mg, 6.77 µmol) and $Pd(PPh_3)_2Cl_2$ (1.98 mg, 2.82 mol) were added and the pale yellow solution was stirred at 45 °C for 41.5 h. TLC (CH_2Cl_2/EtOH, 15:1; R_f of nitroxide **10** = 0.55; R_f of diiodo benzene **11** = 0.33; R_f of NO-NO ruler **12** = 0.10) of the reaction mixture (a brown suspension) indicated an incomplete conversion. Piperidine (500 µL, 5 mmol), CuI (3.15 mg, 16.51 µmol) and $Pd(PPh_3)_4$ (3.64 mg, 3.15 µmol) were added. The solution was stirred at 45 °C for 1 d. Water (15 mL) and CH_2Cl_2 (15 mL) were added and the phases were separated. The aqueous phase was extracted with CH_2Cl_2 (3 x 15 mL). The combined organic phases were washed with aqueous HCl (1 M, 2 x 10 mL) and water (10 mL), dried over Na_2SO_4, and filtered. The solvents of the filtrate were removed. Preparative

HPLC on a Silica(2) column (5 µm, 100 Å, 21.2 mm × 250 mm, Luna® Phenomenex) applying an isocratic elution (CH_2Cl_2/EtOH, 93:7) with a flow rate of 15 mL/min at room temperature and UV detection at 254 nm gave NO-NO ruler **12** (61.7 mg, 35%; retention time: 7.8 – 9.5 min) as a yellow oil. ^{1}H NMR (500 MHz, CD_2Cl_2): All signals are broad and featureless and integrals in the low field region come with a large error. δ [ppm] = 7.90 (br s, 1.8 H, 2 triazole-H), 7.49 (very br s, 2 H, H *meta* to N), 7.23 (br s, 2 H, H *ortho* to OCH_2-triazole), 6.96 (very br s, 4 H, H *ortho* to N), 5.31 (br s, 4 H, $C_{triazole}$-CH_2), 4.49 (br s, 4 H, $N_{triazole}$-CH_2), 4.20 (br s, 6.2 H, 4 CH_2 of PEG), 3.90 bis 3.28 (br s, 102 H, 42 CH_2 and 8 CH_3 of PEG), 2.50 (br s, 2 H, $N_{triazole}$-$CH_2C\underline{H}$). ^{13}C NMR (126 MHz, CD_2Cl_2) *δ* [ppm] = 158.9 ($C_{benzene}$O *ortho* to N), 152.4 ($\underline{C}_{benzene}OCH_2$-triazole), 144.5 (br, $C_{benzene}$N), 142.2 ($\underline{C}_{triazole}CH_2$), 132.4 (br, $C_{benzene}$H *meta* to O), 124.2 ($C_{triazole}$H), 120.3 (br, $C_{benzene}$H *para* to O), 117.9 ($C_{benzene}$H *para* to OCH_2-triazole), 113.8 ($\underline{C}_{benzene}$C≡C-benzene-N), 111.9 and 111.5 ($C_{benzene}$ *para* to N and $C_{benzene}$H *ortho* to N and *ortho* to O), 89.97 and 89.63 ($\underline{C}$≡$\underline{C}$), 71.45, 71.37, 71.35, 70.38, 70.19, 70.07, 70.02, 69.85, 69.79, 69.76, 69.54, 68.77, 68.52, and 67.81 (CH_2 of PEG), 62.8 ($C_{triazole}$-$\underline{C}H_2$), 58.21, 58.16, and 58.09 (OCH_3 of PEG), 48.6 ($N_{triazole}$-$\underline{C}H_2$), 47.0 (br, $\underline{C}H_2$NC=O), 39.97 ($N_{triazole}$-$CH_2\underline{C}H$). MS (ESI) *m/z* = 2235.4 [M + Na + H]$^{2+}$. Accurate MS (ESI) calcd. for [M + 2Na]$^{2+}$ $C_{110}H_{174}N_{10}O_{36}Na_2^{2+}$: 1128.59384; found: 1128.5950.

References

[1] G. Jeschke, M. Sajid, M. Schulte, N. Ramezanian, A. Volkov, H. Zimmermann, A. Godt, *J. Am. Chem. Soc.* **2010**, *132*, 10107.

[2] M. Qi, M. Hülsmann, A. Godt, *J. Org. Chem.* **2016**, *81*, 2549.

[3] I. Ritsch, H. Hintz, G. Jeschke, A. Godt, M. Yulikov, *Phys. Chem. Chem. Phys.* **2019**, *21*, 9810.

[4] H. Wunderer, *Arch. Pharm. Pharm. Med. Chem.* **1973**, *306*, 371.

[5] R. Heathcote, J. A. S. Howell, N. Jennings, D. Cartlidge, L. Cobden, S. Coles, M. Hursthouse, *Dalton Trans.* **2007**, 1309.

[6] D. V. Schoonover, H. W. Gibson, *Tetrahedron Lett.* **2017**, *58*, 242.

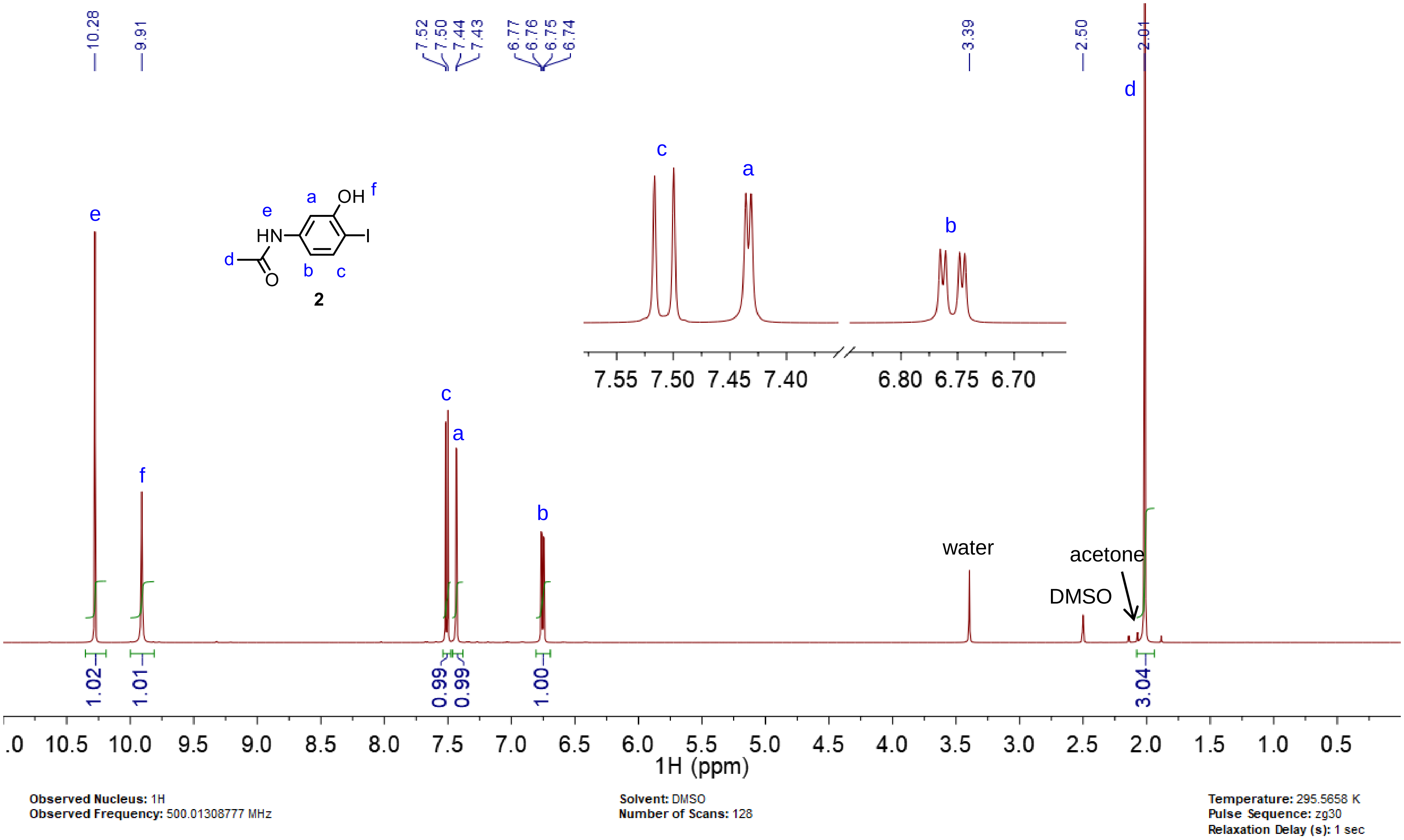

Figure S1. ^{1}H NMR spectrum (500 MHz, DMSO-d_6) of 2-iodo-5-acetaminophenol (**2**).

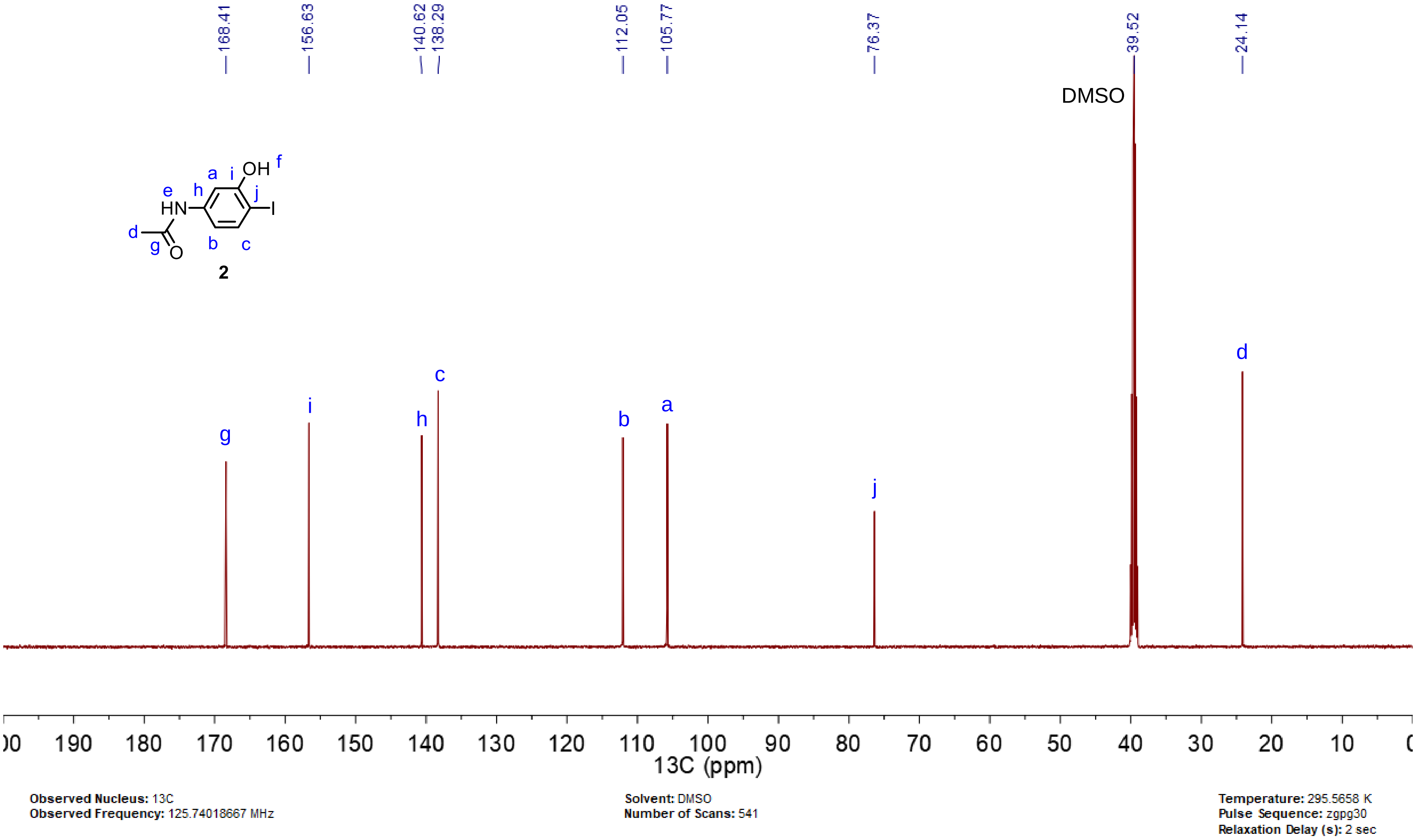

Figure S2. ^{13}C NMR spectrum (126 MHz, DMSO-d_6) of 2-iodo-5-acetaminophenol (**2**).

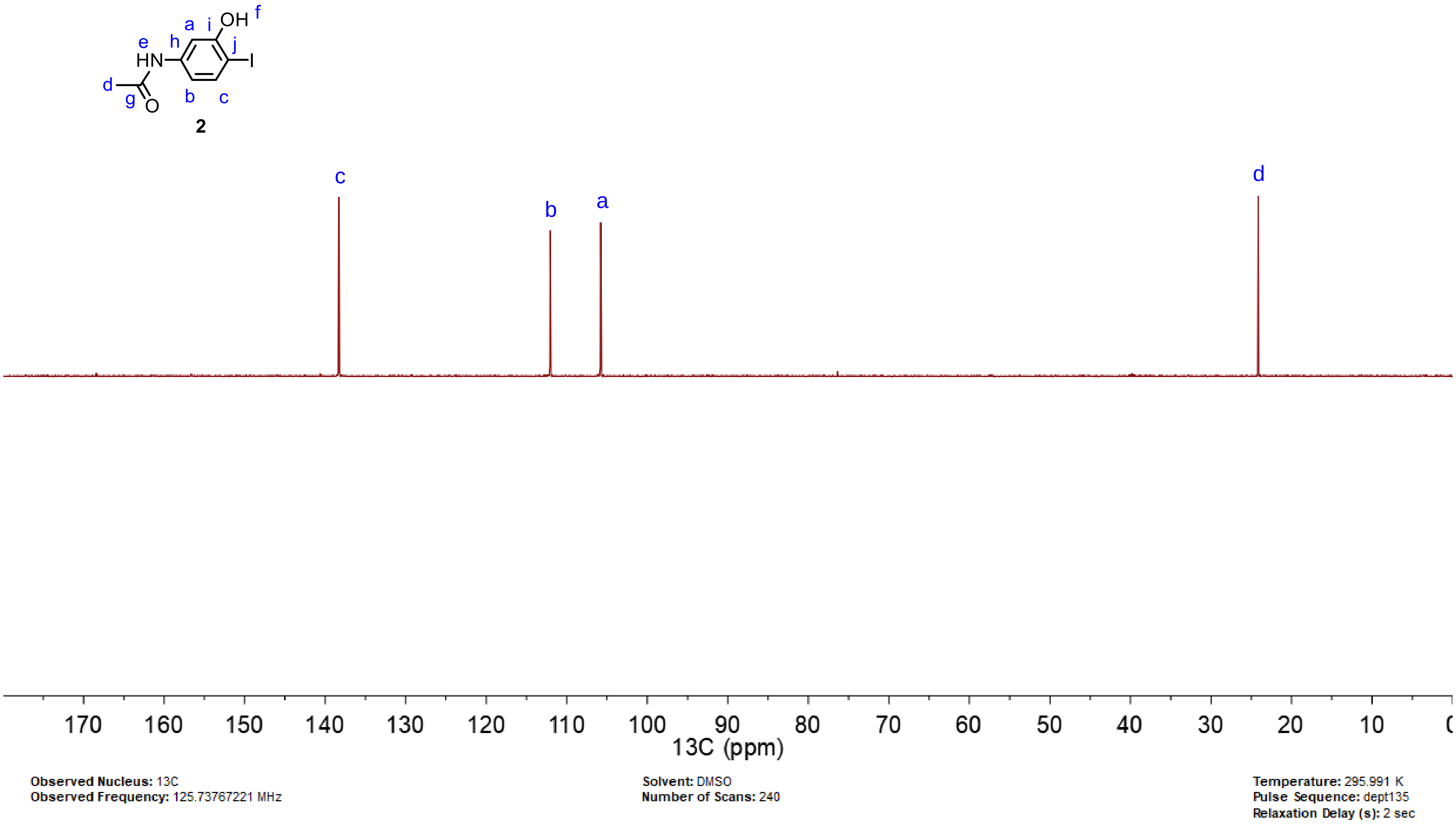

Figure S3. ^{13}C DEPT 135 NMR spectrum (126 MHz, DMSO-d_6) of 2-iodo-5-acetaminophenol (**2**).

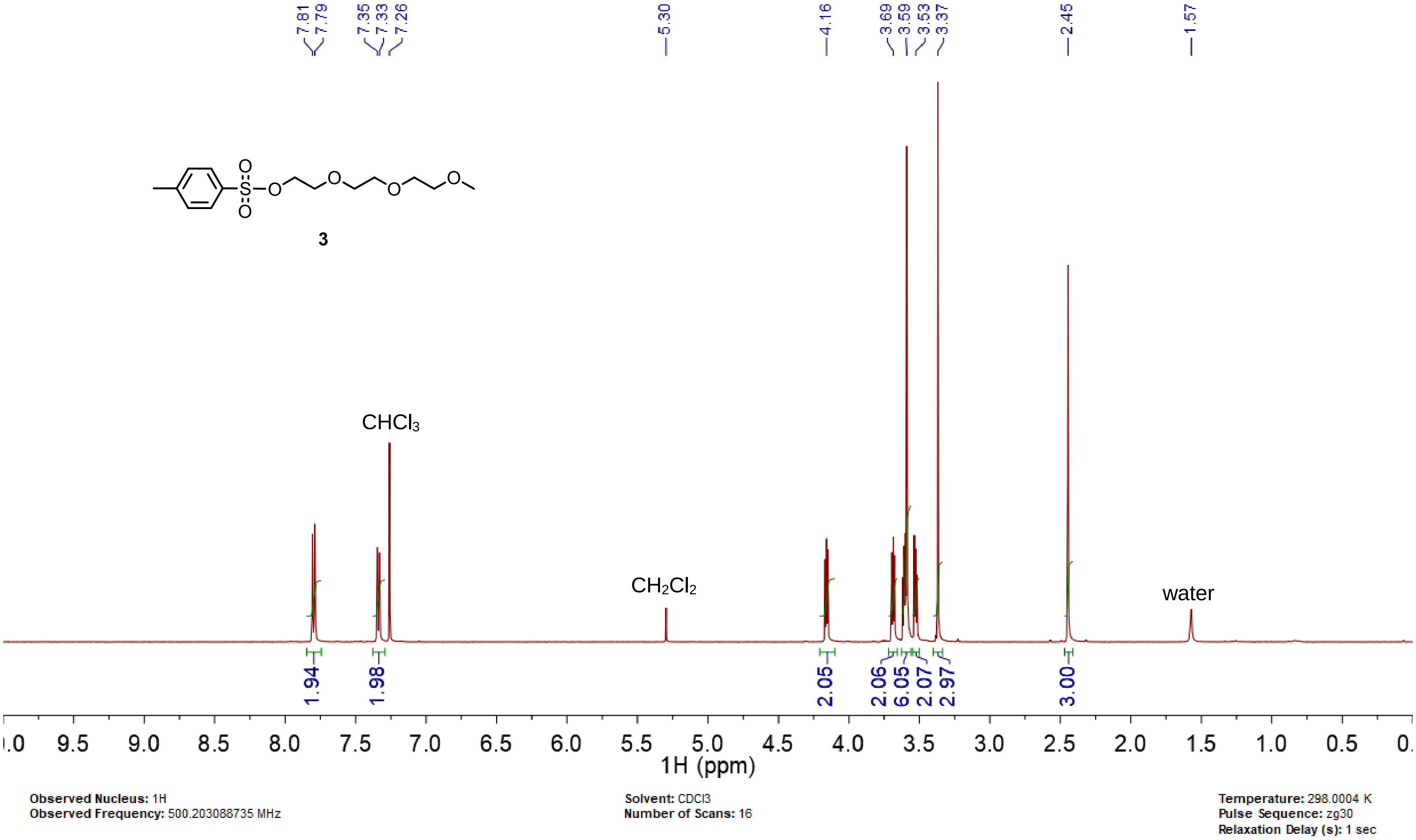

Figure S4. ^{1}H NMR spectrum (500 MHz, $CDCl_3$) of PEG-tosylate **3**.

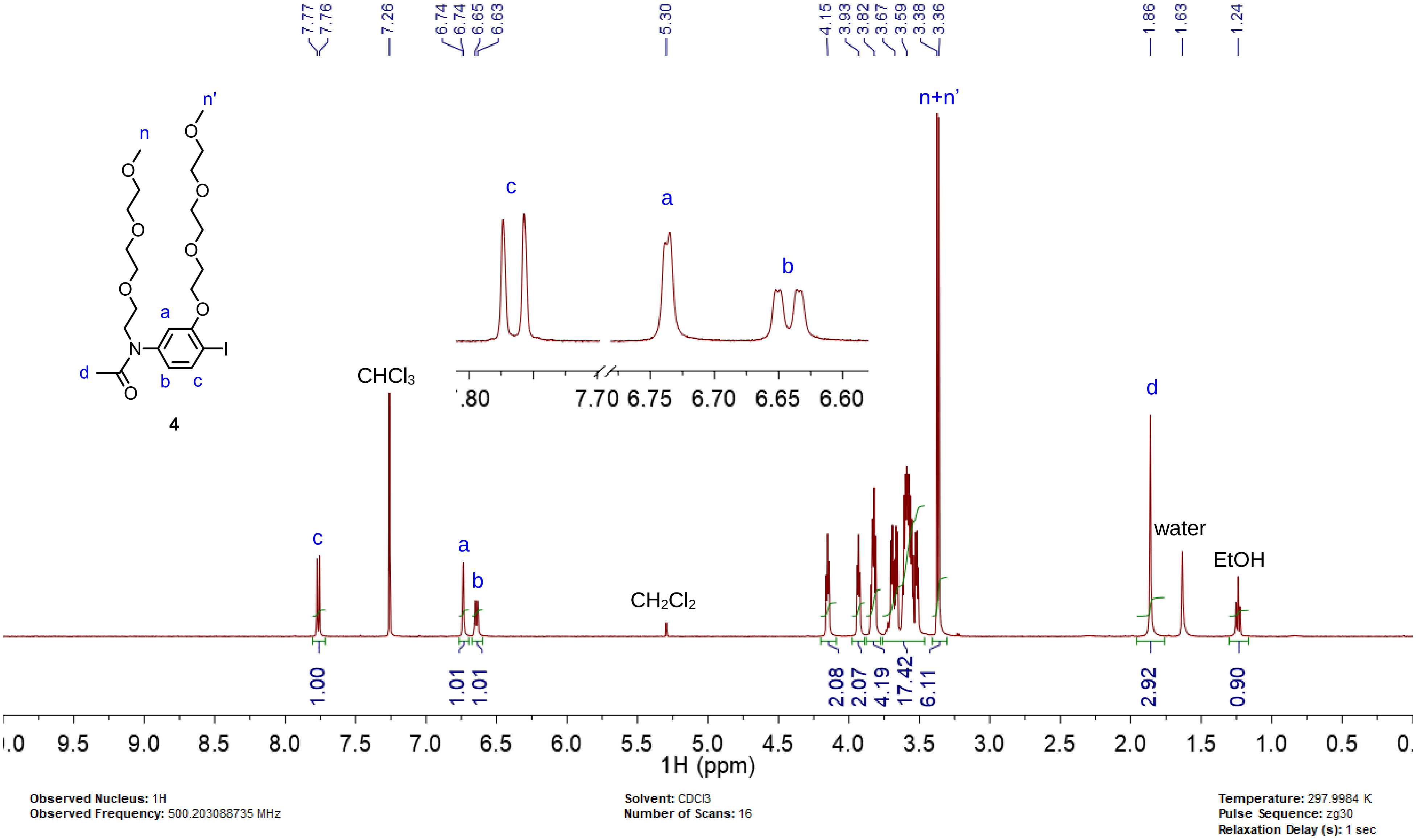

Figure S5. ^{1}H NMR spectrum (500 MHz, $CDCl_3$) of pegylated amide **4**.

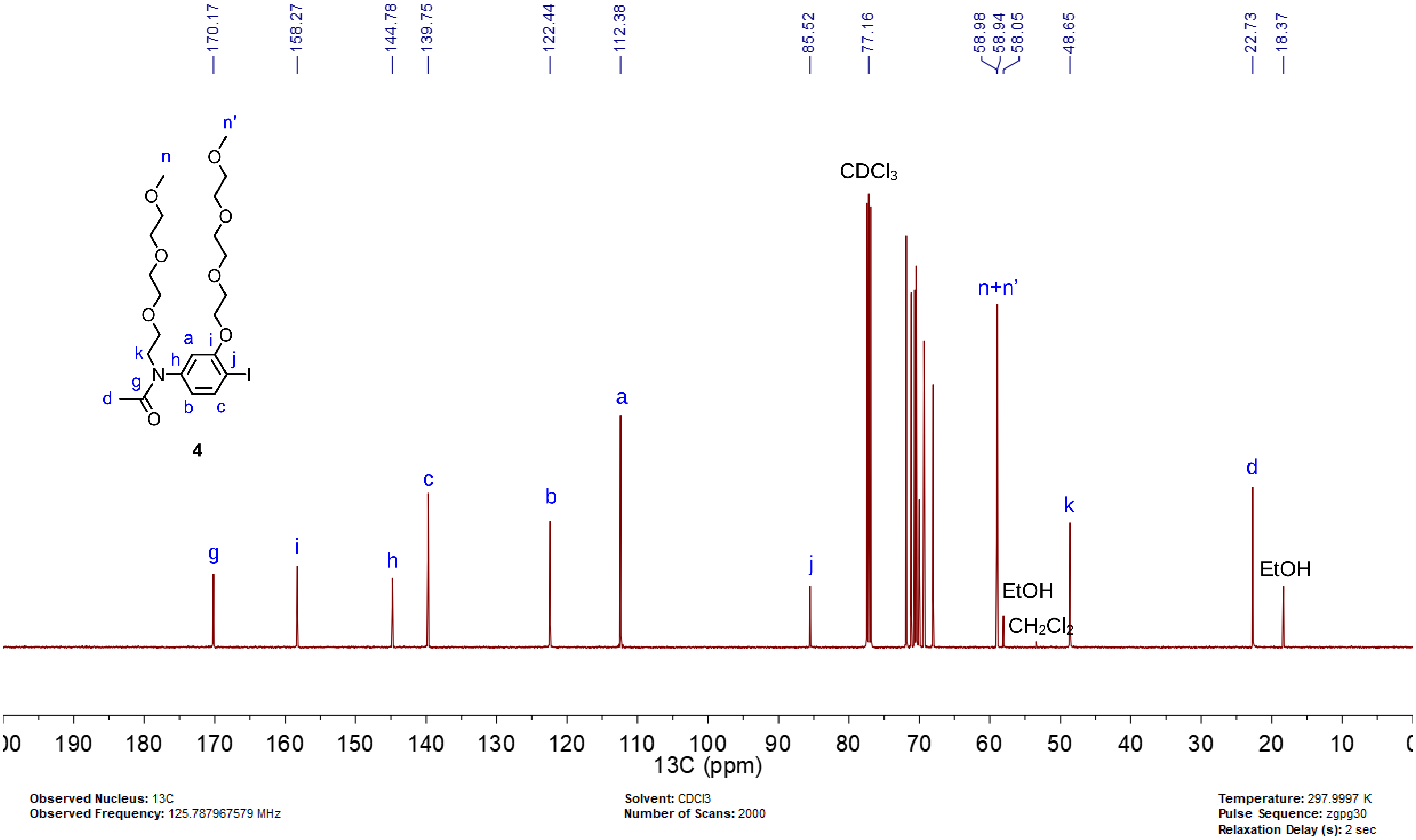

Figure S6. ^{13}C NMR spectrum (126 MHz, $CDCl_3$) of pegylated amide **4**.

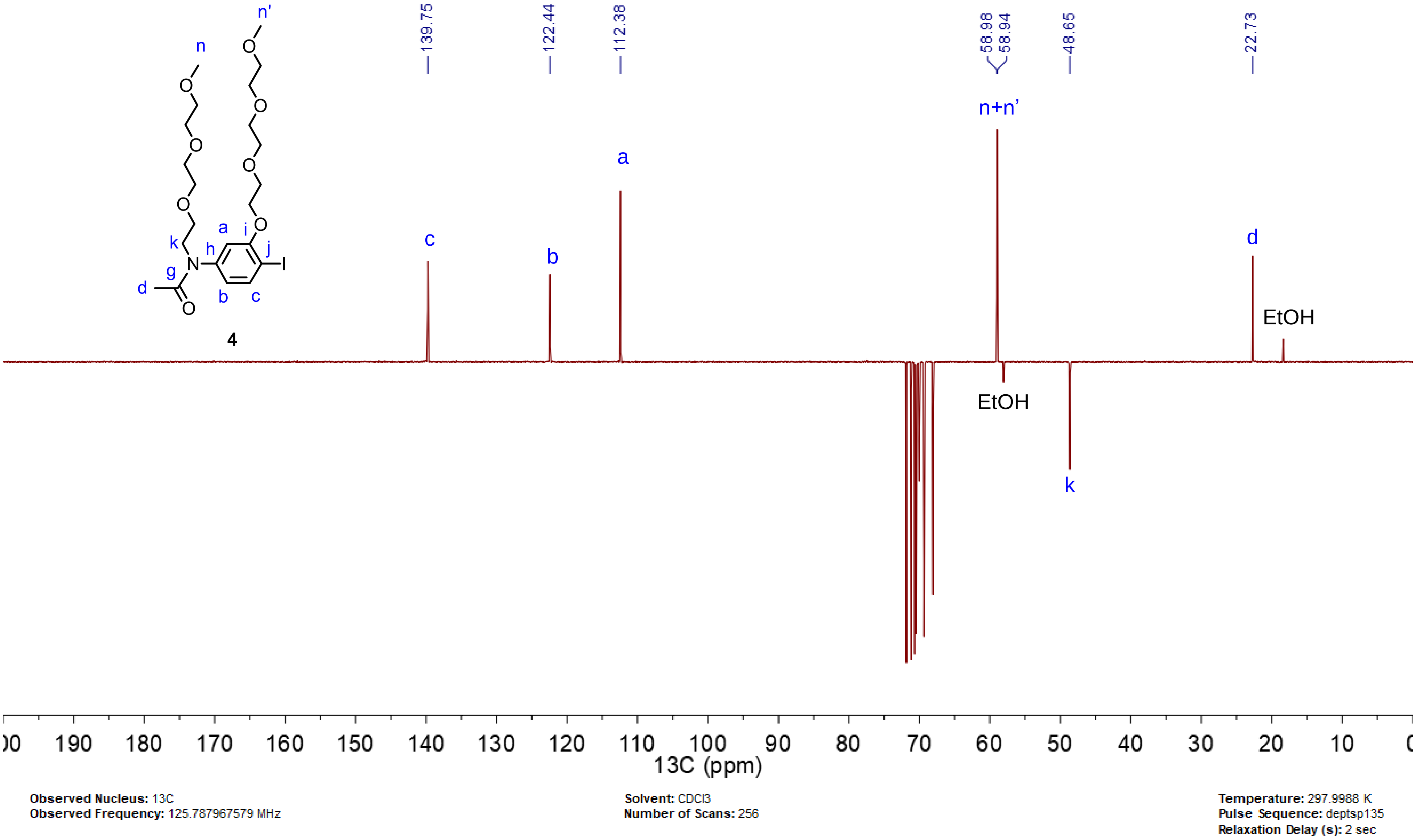

Figure S7. ^{13}C DEPT 135 NMR spectrum (126 MHz, $CDCl_3$) of pegylated amide **4**.

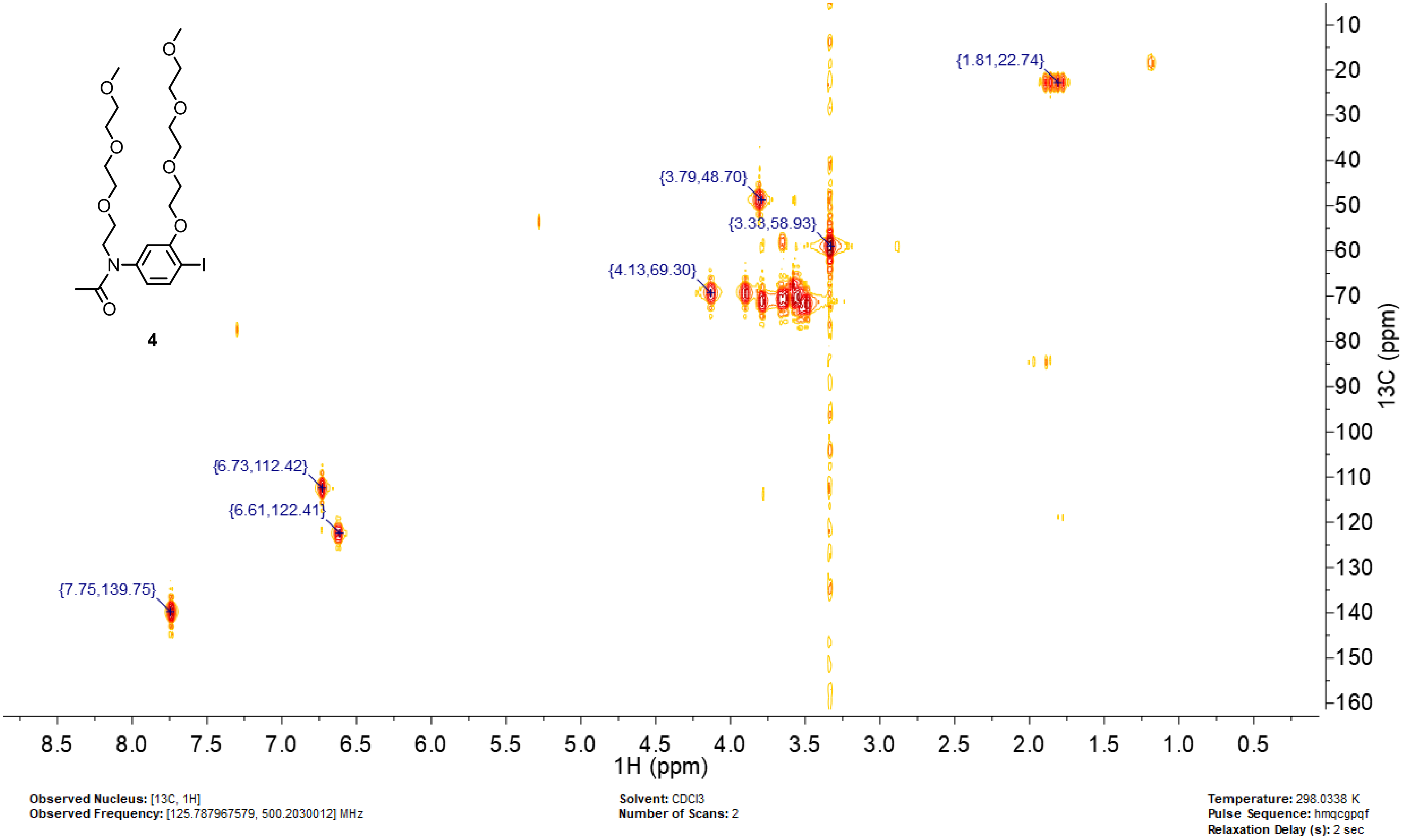

Figure S8. HMQC NMR spectrum (126 MHz, 500 MHz, $CDCl_3$) of pegylated amide **4**.

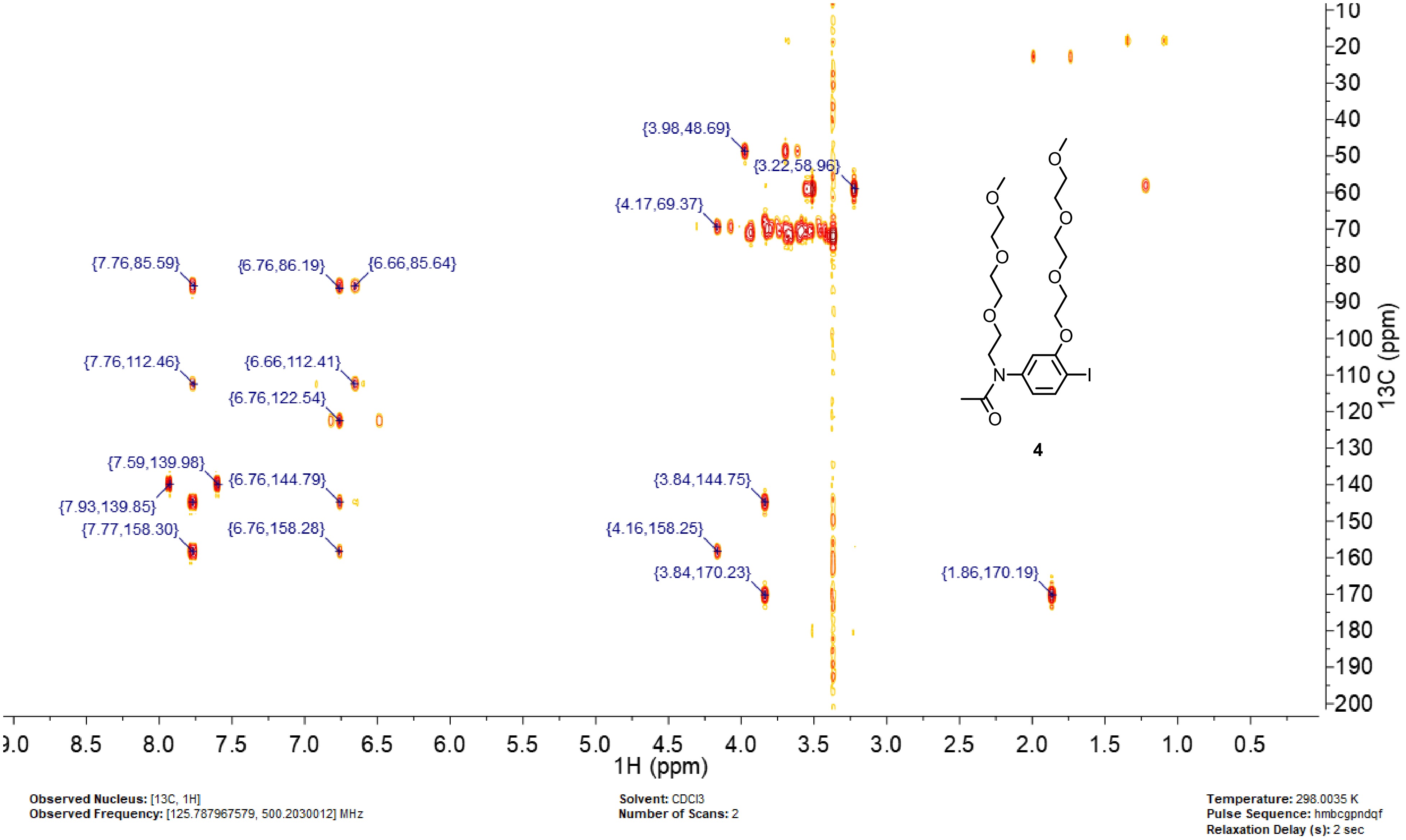

Figure S9. HMBC NMR spectrum (126 MHz, 500 MHz, $CDCl_3$) of pegylated amide **4**.

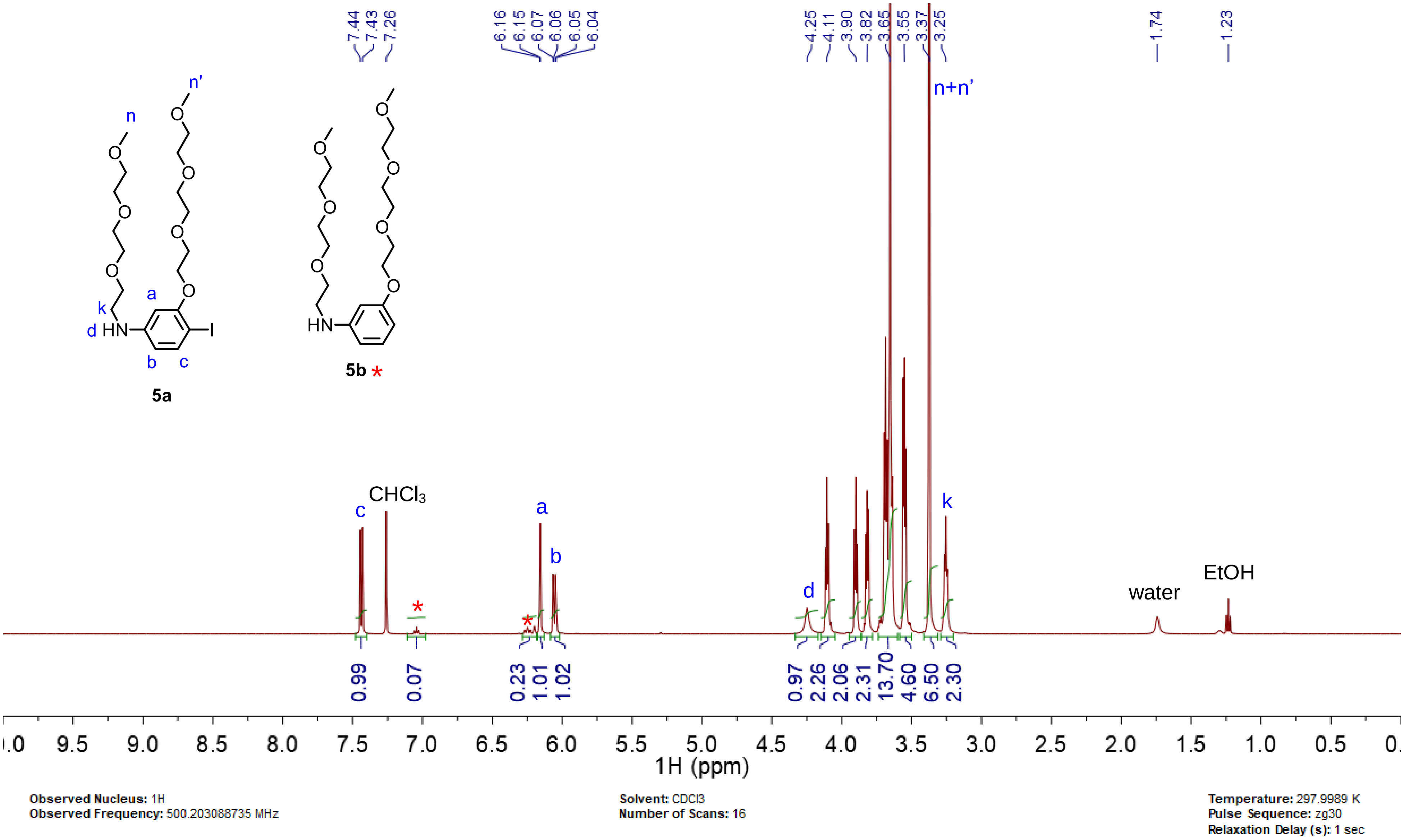

Figure S10. ^{1}H NMR spectrum (500 MHz, $CDCl_3$) of pegylated amine **5a**.

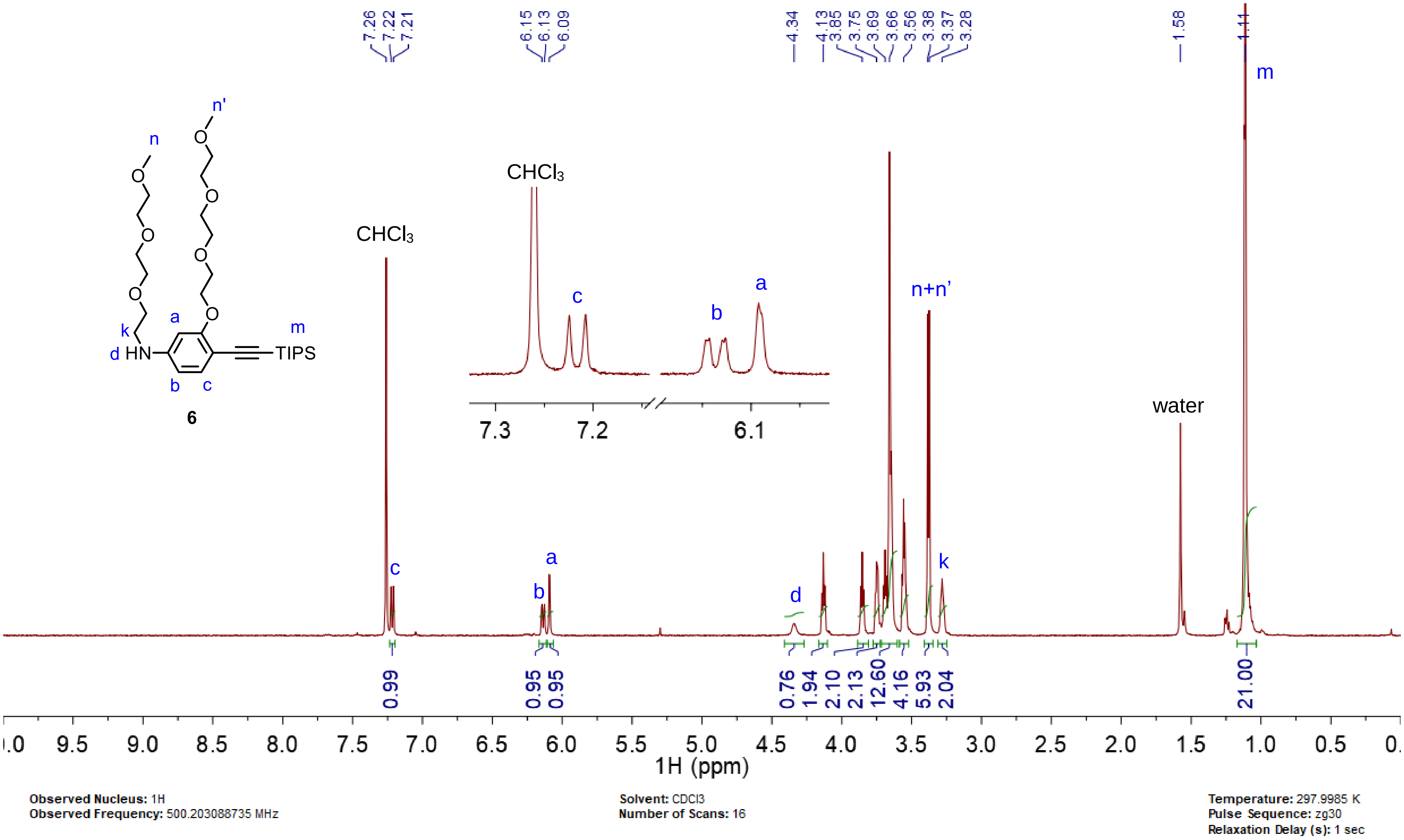

Figure S11. ^{1}H NMR spectrum (500 MHz, $CDCl_3$) of TIPS-protected acetylene **6**.

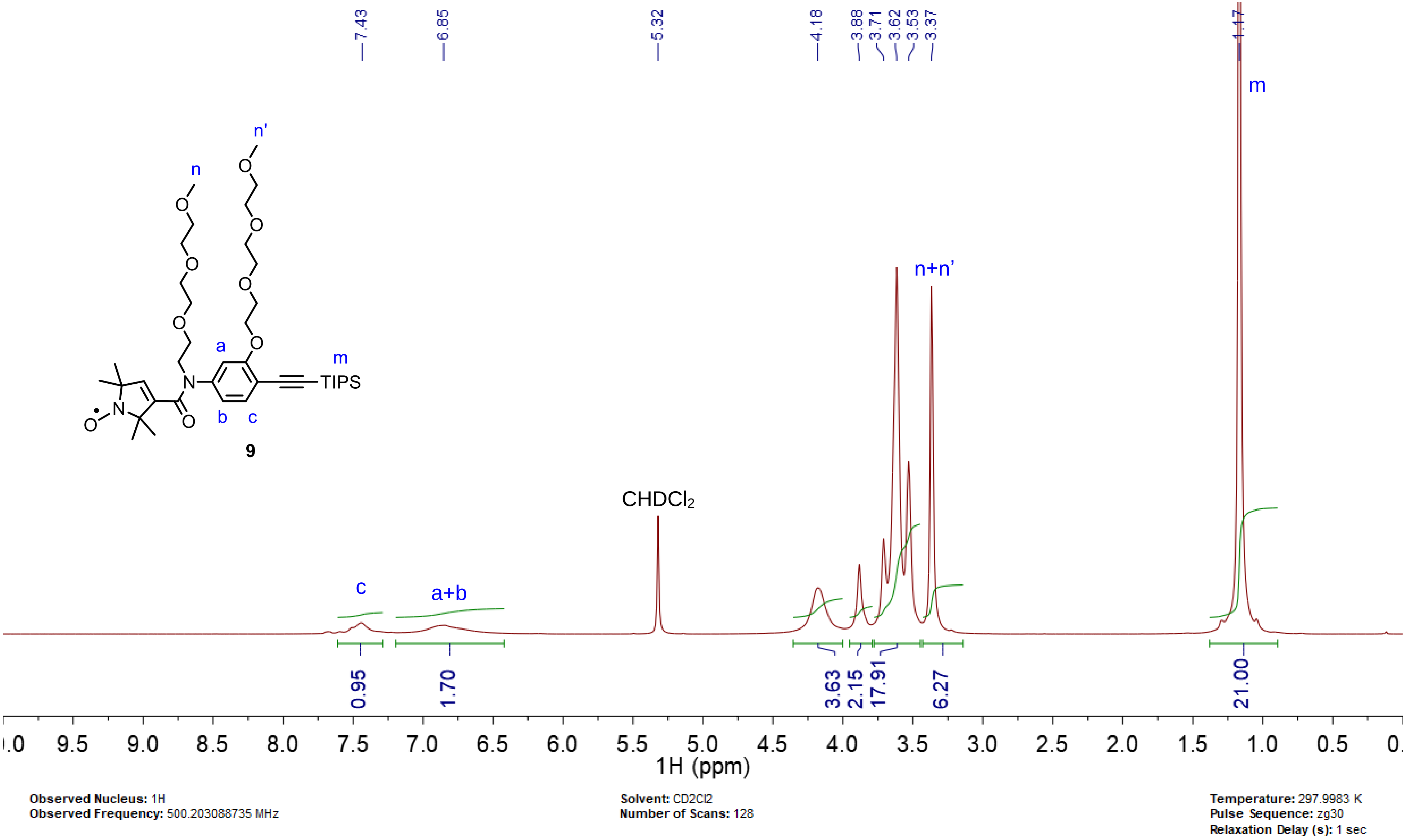

Figure S12. ^{1}H NMR spectrum (500 MHz, $CDCl_3$) of nitroxide **9**.

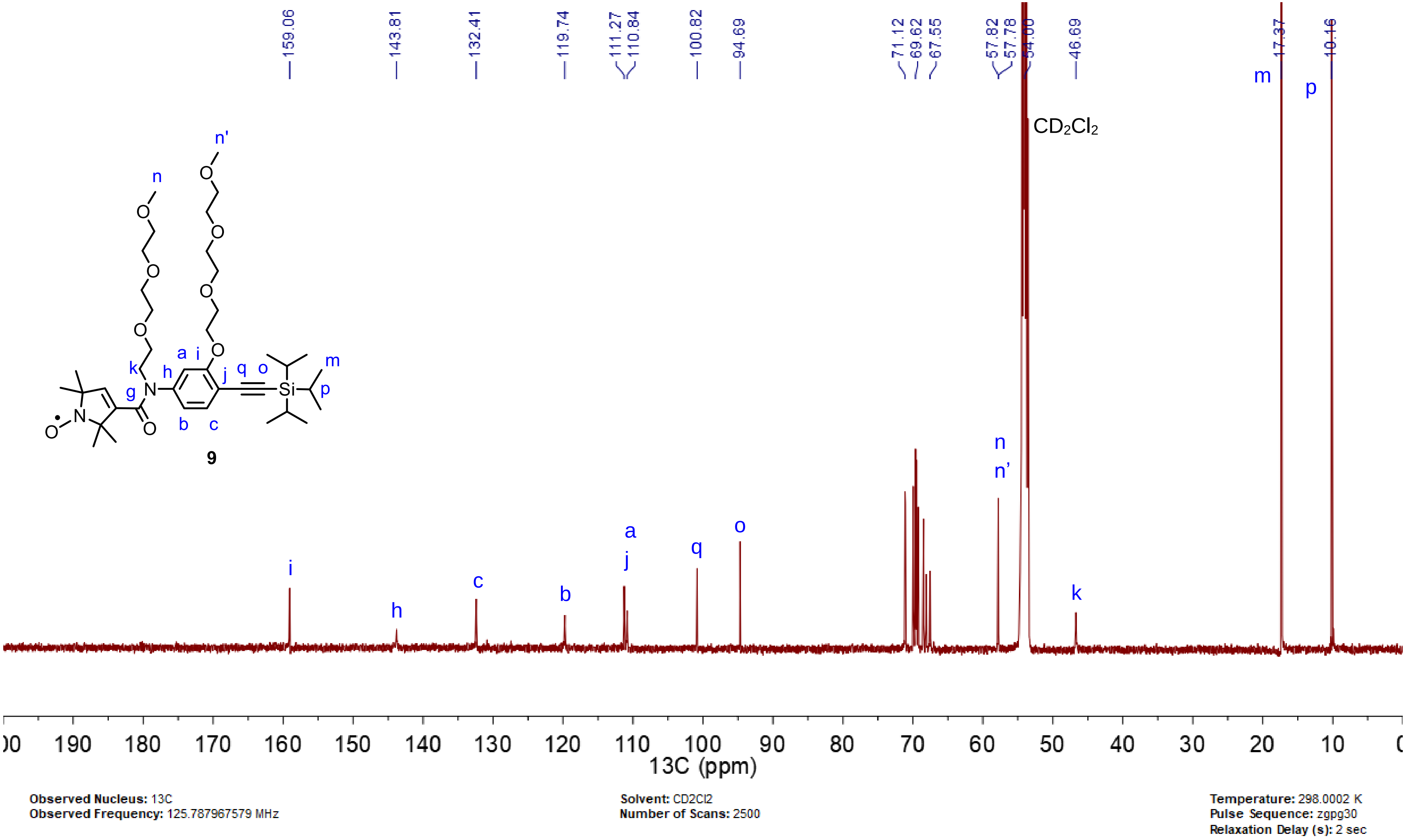

Figure S13. ^{13}C NMR spectrum (126 MHz, $CDCl_3$) of nitroxide **9**.

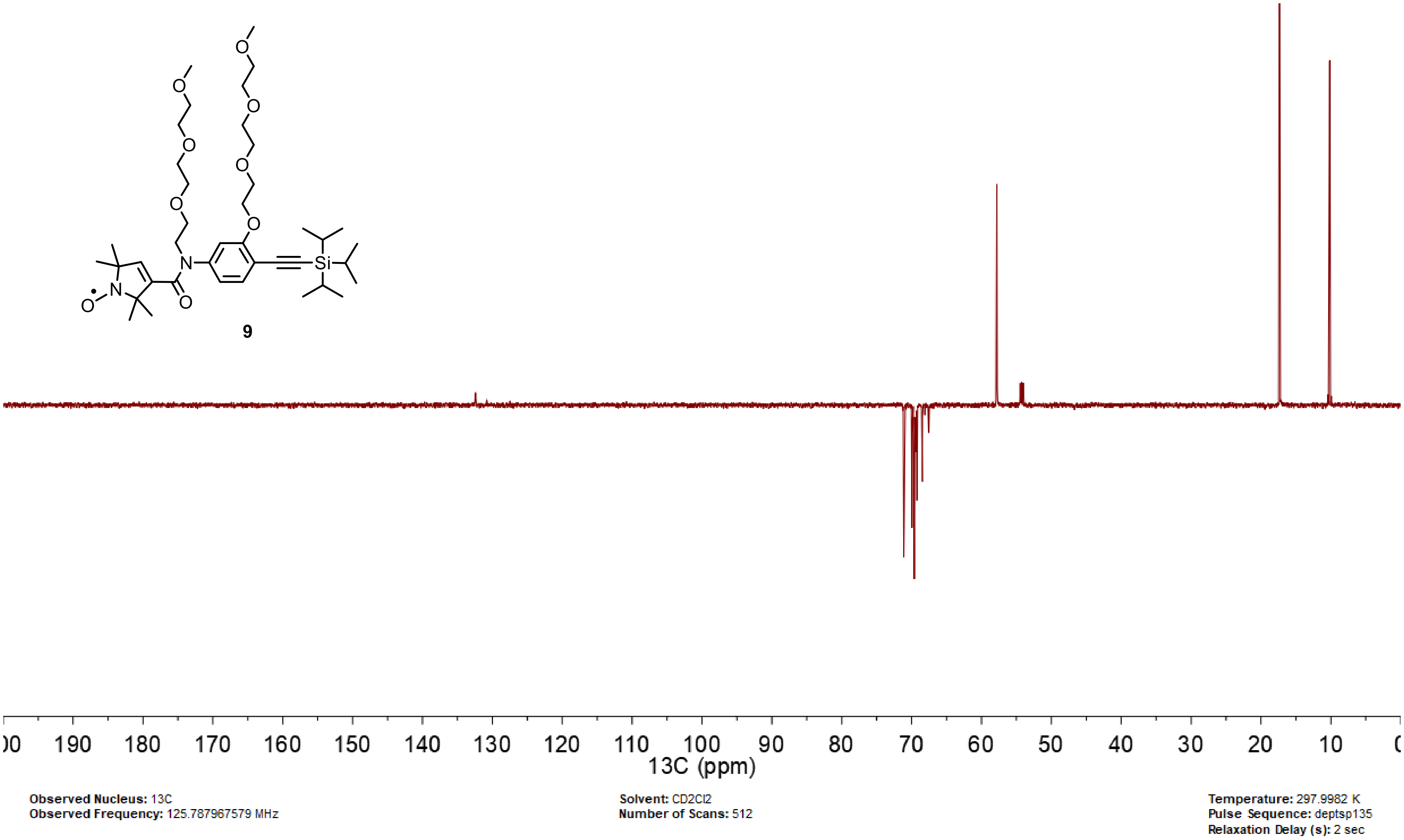

Figure S14. ^{13}C DEPT 135 NMR spectrum (126 MHz, $CDCl_3$) of nitroxide **9**.

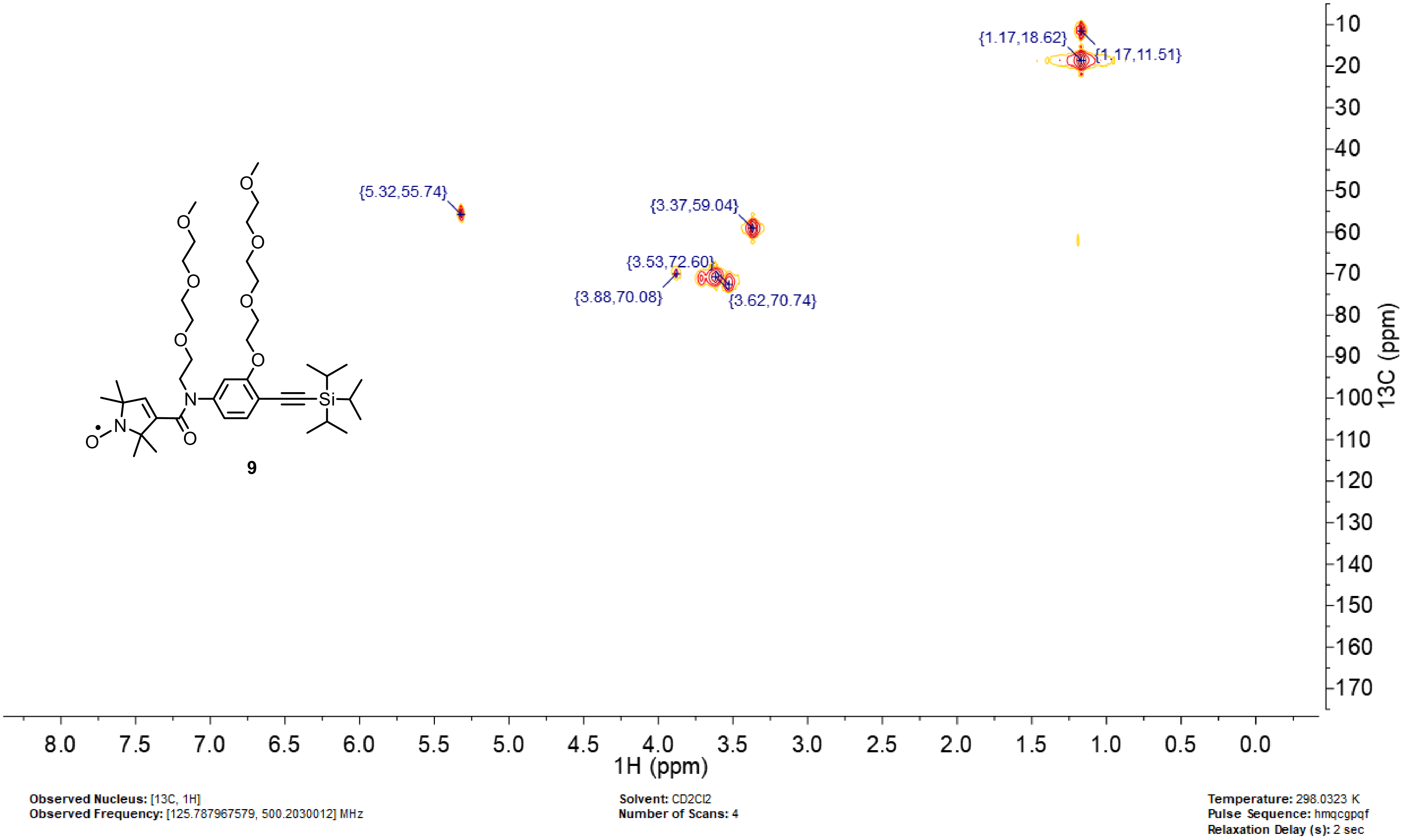

Figure S15. HMQC NMR spectrum (126 MHz, 500 MHz, CD_2Cl_2) of nitroxide **9**.

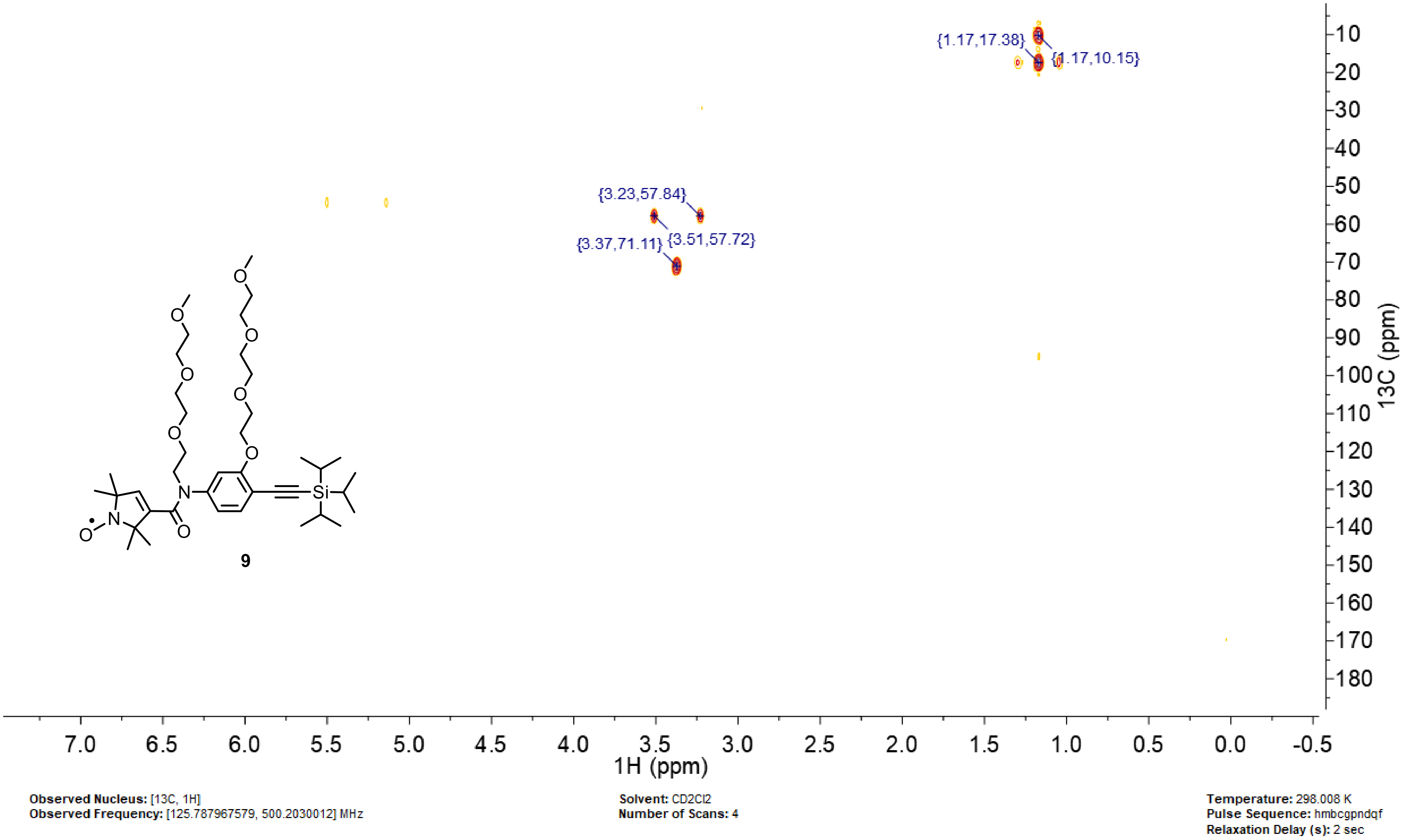

Figure S16. HMBC NMR spectrum (126 MHz, 500 MHz, CD_2Cl_2) of nitroxide **9**.

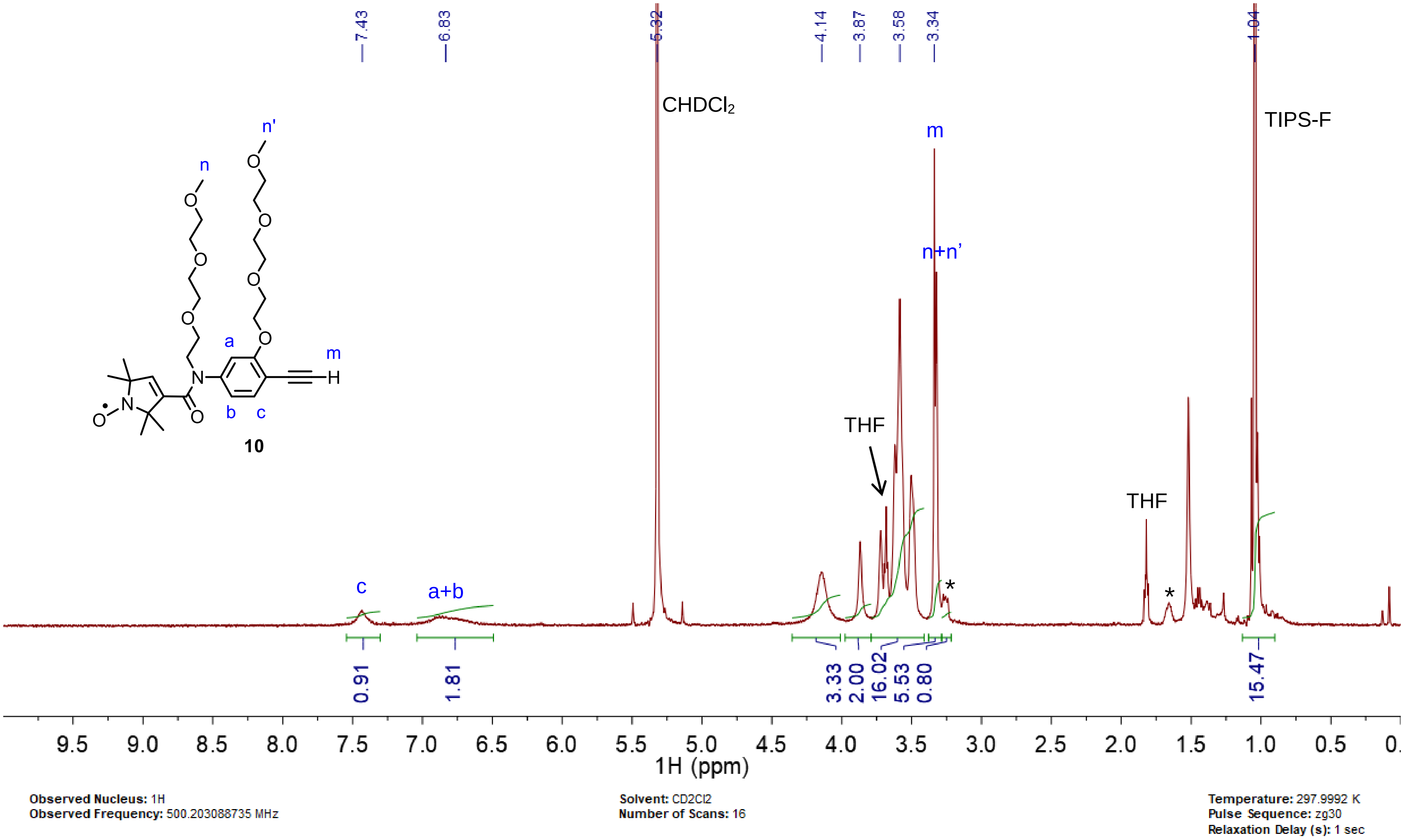

Figure S17. ^{1}H NMR spectrum (500 MHz, CD_2Cl_2) of alkyne **10**. * Bu_4NY (Y = F, OH).

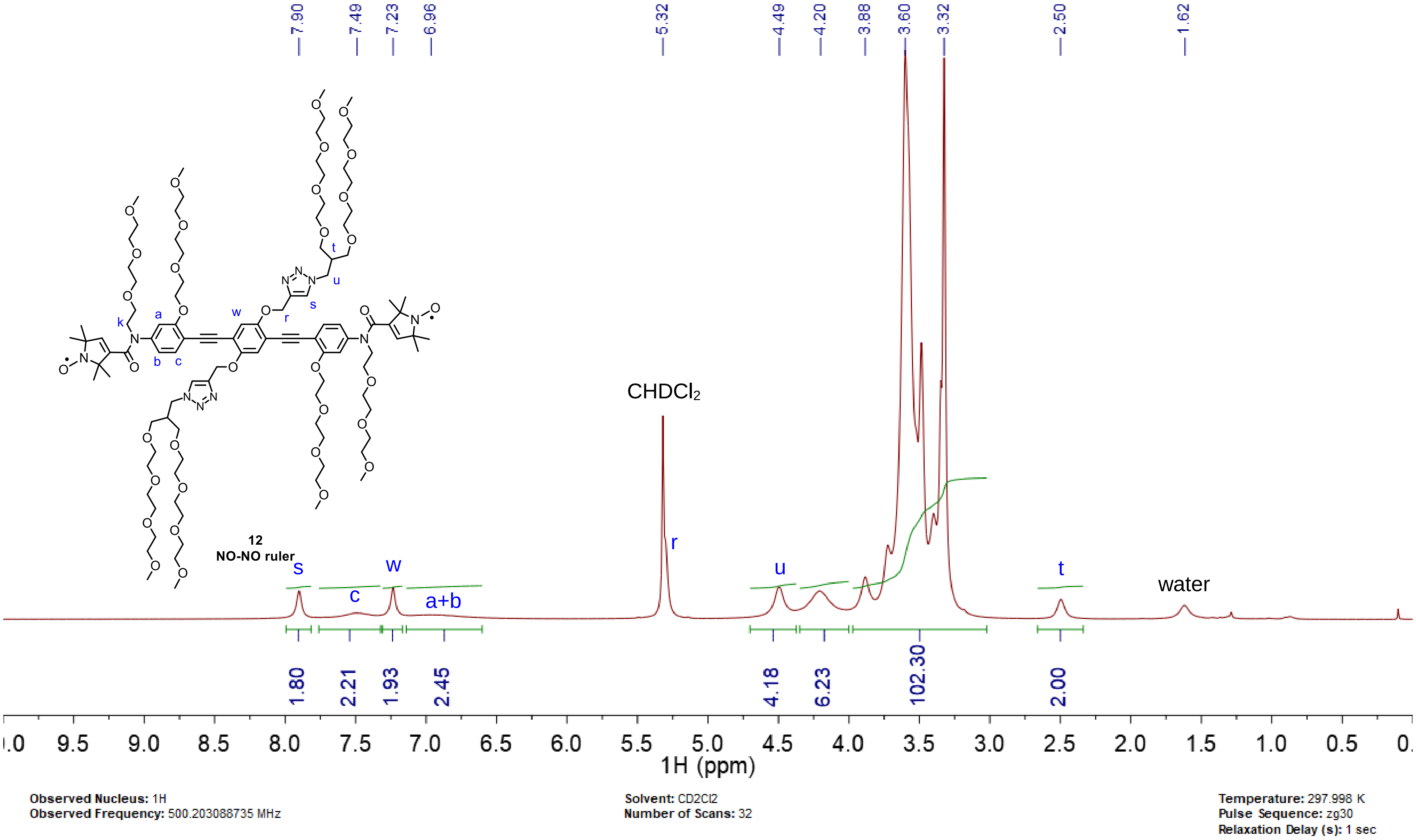

Figure S18. ^{1}H NMR spectrum (500 MHz, CD_2Cl_2) of NO-NO ruler **12**.

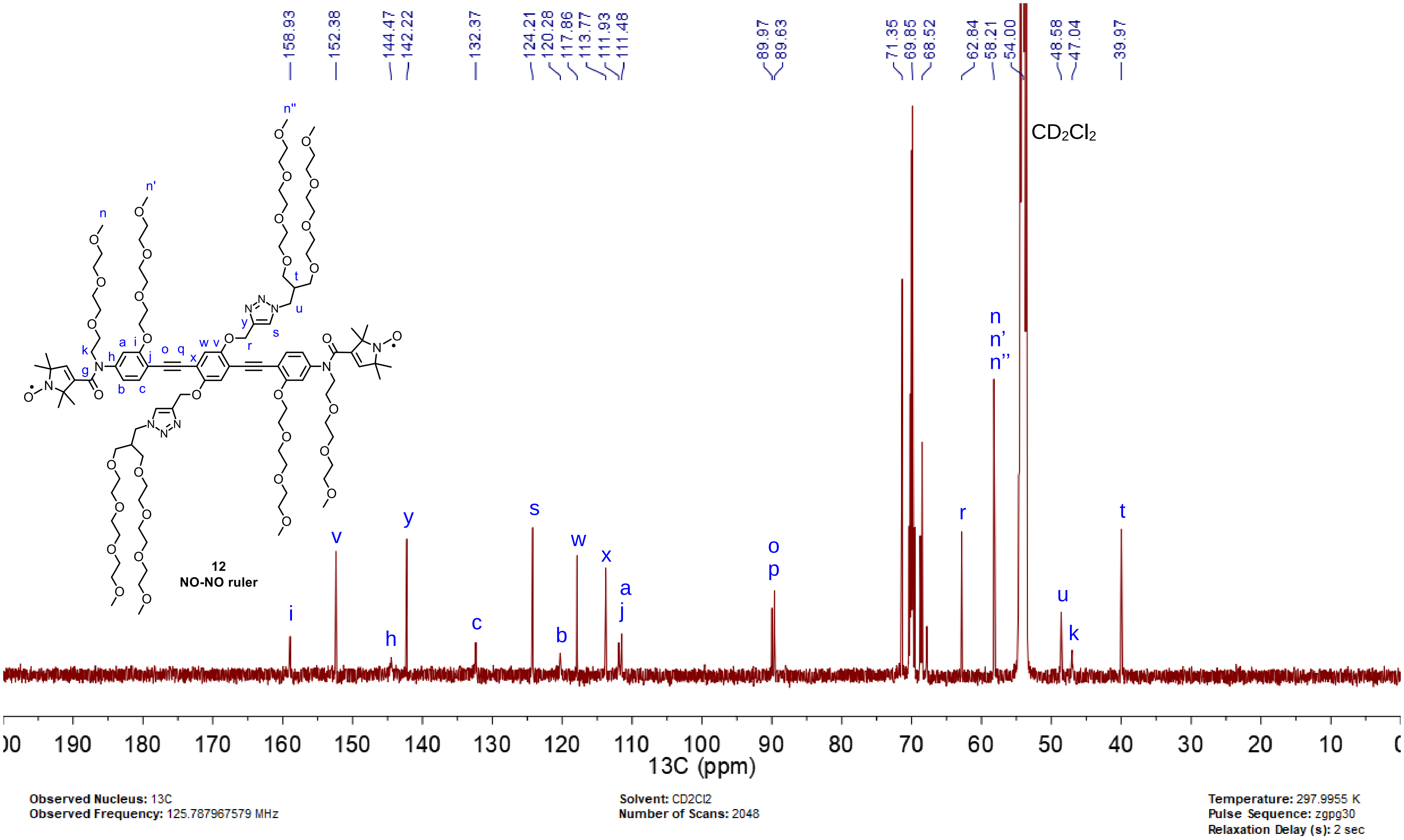

Figure S19. ^{13}C NMR spectrum (126 MHz, CD_2Cl_2) of NO-NO ruler **12**.

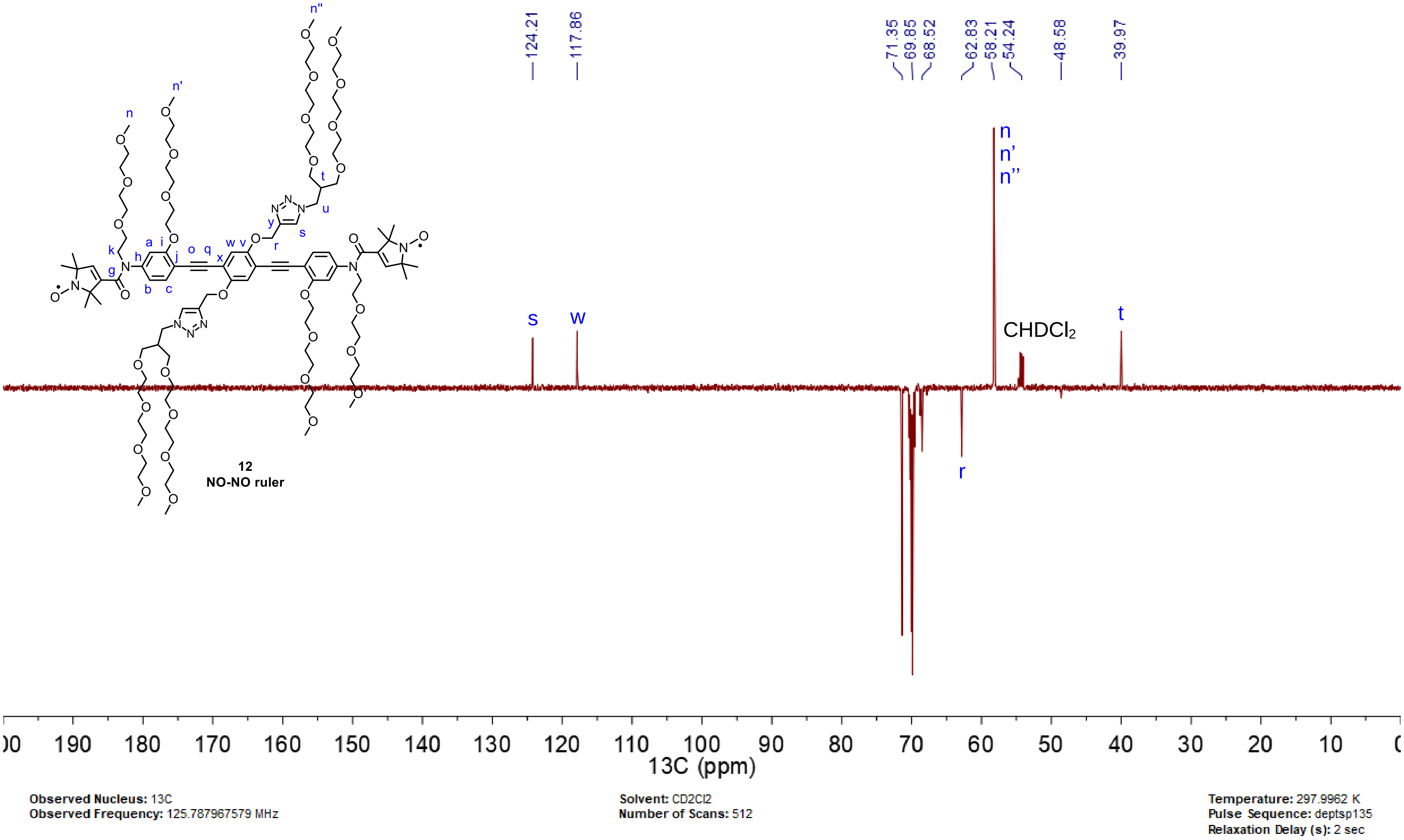

Figure S20. ^{13}C DEPT 135 NMR spectrum (126 MHz, CD_2Cl_2) of NO-NO ruler **12**.

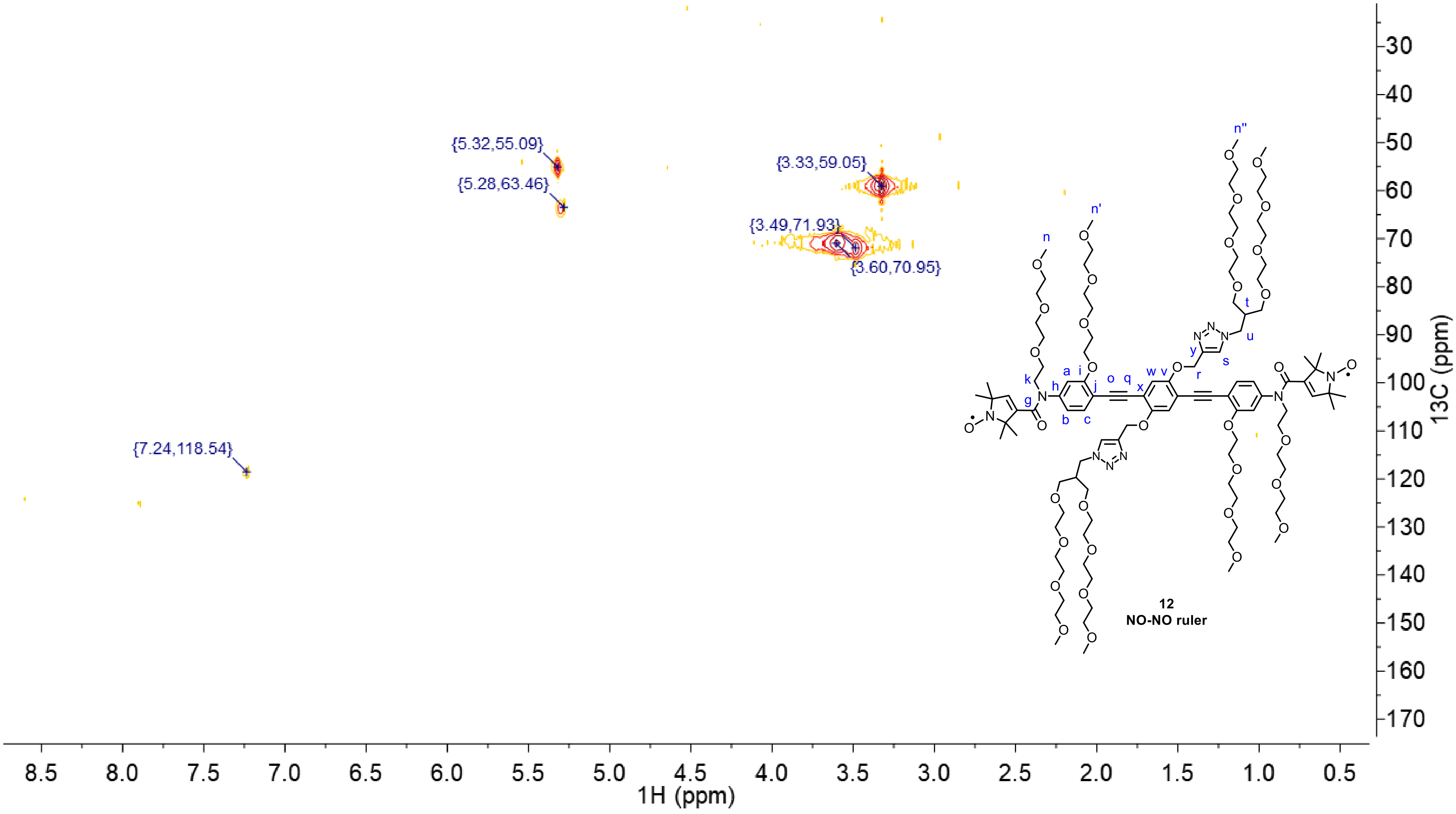

Observed Nucleus: [13C, 1H]
Observed Frequency: [125.787967579, 500.2030012] MHz

Solvent: CD2Cl2
Number of Scans: 4

Temperature: 298.0338 K
Pulse Sequence: hmqcgpqf
Relaxation Delay (s): 2 sec

Figure S21. HMQC NMR spectrum (126 MHz, 500 MHz, CD_2Cl_2) of NO-NO ruler **12**.

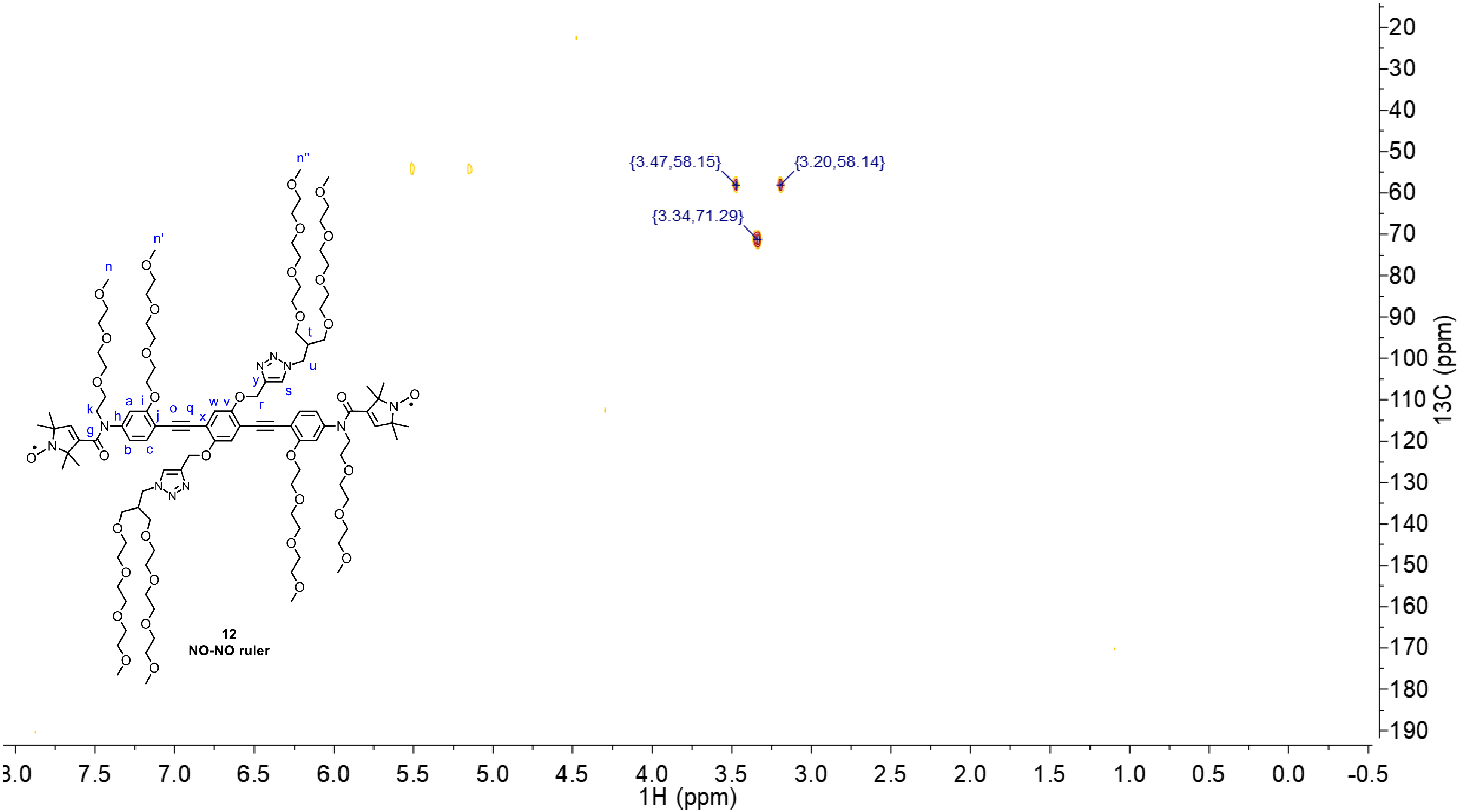

Observed Nucleus: [13C, 1H]
Observed Frequency: [125.787967579, 500.2030012] MHz

Solvent: CD2Cl2
Number of Scans: 4

Temperature: 298.004 K
Pulse Sequence: hmbcgpndqf
Relaxation Delay (s): 2 sec

Figure S22. HMBC NMR spectrum (126 MHz, 500 MHz, CD_2Cl_2) of NO-NO ruler **12**.

SI part B

EPR

Contents

1 Sample preparation **2**

2 Nutation experiments **2**

3 Relaxation **3**
3.1 Longitudinal relaxation 3
3.2 Transverse relaxation 4
3.3 Optimization of the swapped NOGd DEER setup 5

4 DEER data **6**
4.1 Gaussian versus Tikhonov analysis 6
4.2 Quantification 6
4.3 Equimolar ruler mixtures 8
4.3.1 Sample preparation 8
4.3.2 DEER data 9
4.4 Spectrometer artifact 12
4.5 DeerNet 13
4.6 Crosstalk signal overview 14
4.7 Crosstalk identification and suppression 15

1 Sample preparation

sample	ruler	V_{stock} [µl]	$c_{stock(spin)}$ [µM]	$V_{d8\text{-}glycerol}$ [µl]	V_{final} [µl]	$c_{final(spin)}$ [µM]
**NO-NO**	NO-NO<br>NO-Gd<br>Gd-Gd	20<br>-<br>-	100<br>-<br>-	20	40	50<br>-<br>-
**NO-Gd**	NO-NO<br>NO-Gd<br>Gd-Gd	-<br>20<br>-	-<br>100<br>-	20	40	-<br>50<br>-
**Gd-Gd**	NO-NO<br>NO-Gd<br>Gd-Gd	-<br>-<br>20	-<br>-<br>200	20	40	-<br>-<br>100
**NO-NO + NO-Gd**	NO-NO<br>NO-Gd<br>Gd-Gd	10<br>10<br>-	100<br>100<br>-	20	40	25<br>25<br>-
**NO-NO + 2xGd-Gd**	NO-NO<br>NO-Gd<br>Gd-Gd	10<br>-<br>10	100<br>-<br>200	20	40	25<br>-<br>50
**NO-Gd + 2xGd-Gd**	NO-NO<br>NO-Gd<br>Gd-Gd	-<br>10<br>10	-<br>100<br>200	20	40	-<br>25<br>50
**NO-NO + NO-Gd + 2xGd-Gd**	NO-NO<br>NO-Gd<br>Gd-Gd	6.67<br>6.67<br>6.67	100<br>100<br>200	20	40	16.67<br>16.67<br>33.33

Table S1: Sample preparation of the NO-NO, NO-Gd and Gd-Gd rulers in a 1:1:2 ratio. Stock solutions of NONO, NOGd and GdGd rulers were prepared in water using spin counting information obtained from X-band continous wave (cw) EPR for nitroxide (NO) and Q-band field-swept echo (FSE) detected spectra for gadolinium (Gd) (data not shown). 50% v/v (20 µl) fully deuterated glycerol was added to all samples as cryoprotectant. The DEER data obtained on these samples are presented in Fig. 4 - 8 in the main text.

2 Nutation experiments

(a) pulses at spectral maximum of Gd

AWG amplitude [%]		10	20	30	40	50	60	70	80	90	100
π-pulse length [ns] at	12 dB	126	72	53	40	37	34	31	**30**	29	28
	6 dB	70	41	31	**28**	27	26	25	24	-	-
	0 dB	39	**27**	25	11	11	-	-	-	-	-

(b) pulses at the spectral maximum of NO

AWG amplitude [%]		10	20	30	40	50	60	70	80	90	100
π-pulse length [ns] at	12 dB	140	76	56	45	40	35	33	**32**	31	30
	6 dB	-	-	-	**29**	-	-	-	-	-	-
	0 dB	-	**30**	-	-	-	-	-	-	-	-

Table S2: Power dependence of Gd(III) π-pulses (related to Fig. 2 in the main text). Transient nutation experiments were performed with the pulses set at two different spectral positions in the NO-Gd ruler sample at 10 K. Data were recorded at 12, 6 and 0 dB attenuation, with variable AWG amplitudes. Shot repetition time (srt) was set to 1000 µs, to saturate the NO spins. π-pulse lengths were obtained by fitting the first minimum of each nutation transient shown in Fig. 2.

3 Relaxation

3.1 Longitudinal relaxation

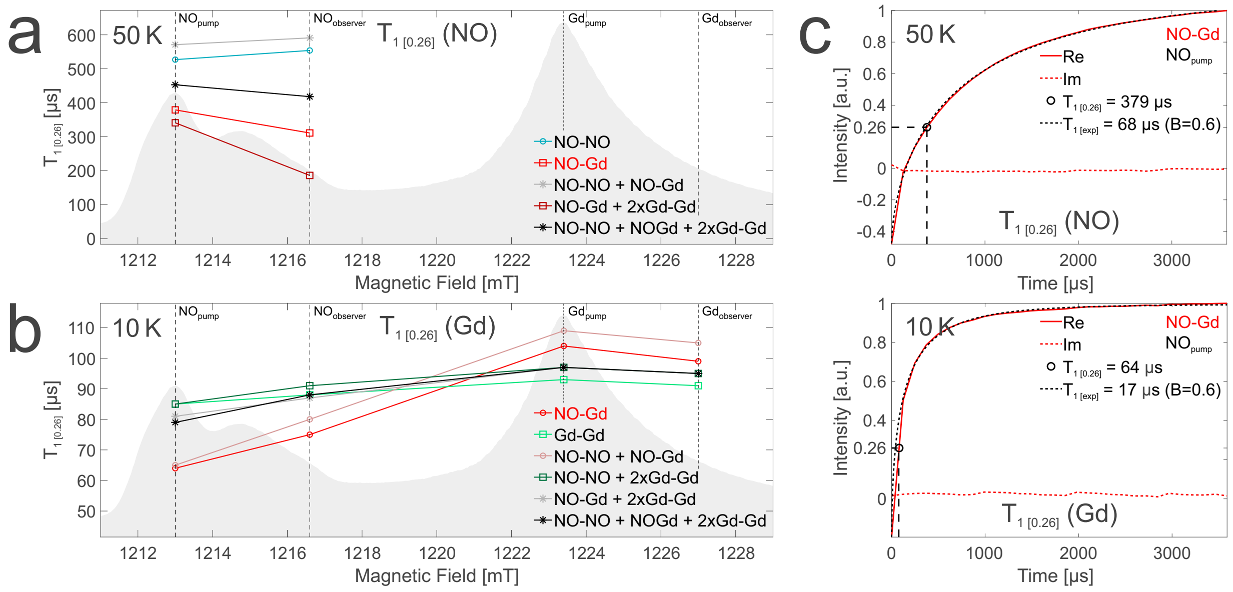

Figure S1: Summary of the longitudinal relaxation data for Gd and NO. To enable comprison between all samples (shown in Table S3), the data were evaluated by determining the time T ($=T_{1\,[0.26]}$) at which the inversion recovery echo signal has an intensity of 0.26 (for details see section 2.2.4 in the main text). We estimate an error of about 5% based on variations when doing independent repetitions. For $T_{1\,[0.26]}$ longer than 0.35 ms the error increases to 20% due to the limited length (3.5 ms) of the inversion recovery traces detected. (a-b) The $T_{1\,[0.26]}$ relaxation times (listed in Table S3) are plotted in correspondence with the positions of the pulses to illustrate the changes. The spectrum of the NO-Gd ruler is shown as a shaded gray area. (a) Inversion recovery experiments performed on the NO pump and observer position at 50 K using a shot repetition time (srt) of 1,000 µs. (b) Inversion recovery experiments on Gd performed at four different positions at 10 K using an srt of 1,000 µs. The selected srt allows to saturate the NO at this temperature, thereby being selective for Gd. (c) Exemplary plots of inversion recovery traces recorded at the NO pump position of the NO-Gd ruler sample. The dashed line show the extracted $T_{1\,[0.26]}$. A stretched exponential fitting of the data is also shown (dotted lines) with the exponent B and the resulting $T_{1\,[exp]}$.

sample	T [K]	$T_{1\,[0.26]}$ (NO) [µs] Magnetic Field Position		$T_{1\,[0.26]}$ (Gd) [µs] Magnetic Field Position			
		NO_{pump}	NO_{obs}	NO_{pump}	NO_{obs}	Gd_{pump}	Gd_{obs}
**NO-NO**	10						
	50	530	500				
**NO-Gd**	10			64	75	104	99
	50	380	310				
**Gd-Gd**	10			85	88	93	91
	50						
**NO-NO** + **NO-Gd**	10			65	80	109	105
	50	570	590				
**NO-NO** + **2xGd-Gd**	10			85	91	97	95
	50						
**NO-Gd** + **2xGd-Gd**	10			81	87	97	95
	50	340	190				
**NO-NO** + **NO-Gd** + **2xGd-Gd**	10			79	88	97	95
	50	450	420			1	

Table S3: Longitudinal $T_{1\,[0.26]}$ relaxation data obtained on all samples investigated at different temperatures. A plot of the data is presented in Fig. S1. We estimate an error of 5% based on variations when doing independent repetitions. For $T_{1\,[0.26]}$ longer than 0.35 ms the error increases to 20% due to the limited length (3.5 ms) of the inversion recovery traces detected (due to AWG memory limitations).

3.2 Transverse relaxation

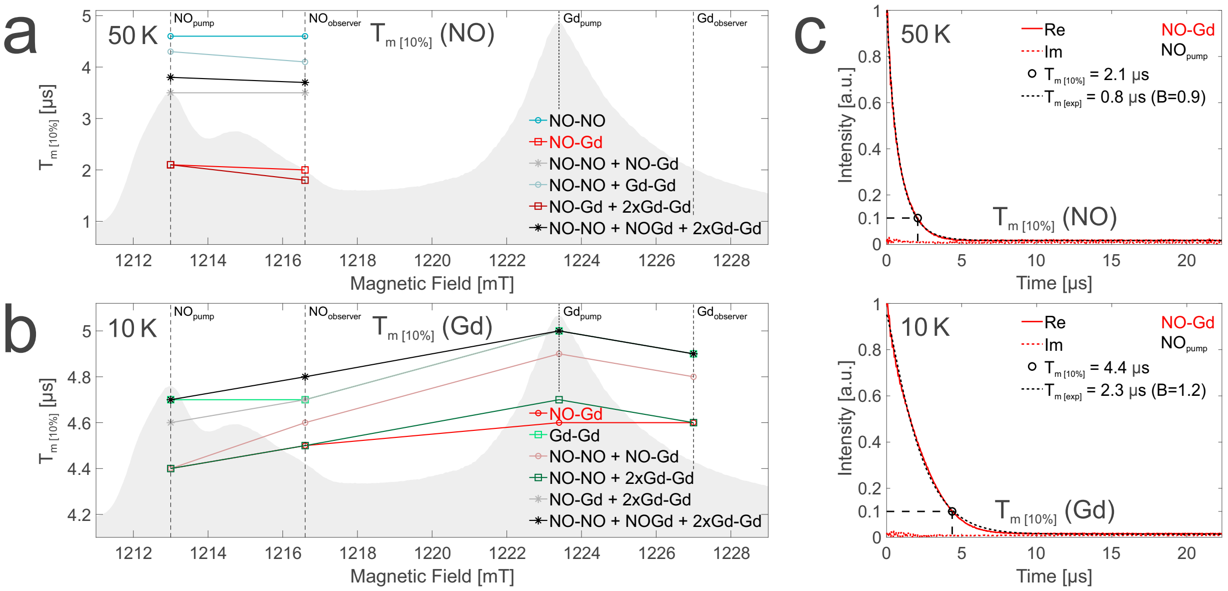

Figure S2: Summary of transverse relaxation data.To enable comprison between all samples (shown in Table S4), the data were evaluated by determining the time ($= T_{m\,[10\%]}$) at which the normalized echo signal has an intensity of 0.10. We estimate an error of about 10% based on variations when doing independent repetitions. (a-b) The $T_{m\,[10\%]}$ relaxation times (listed in Table S4) are plotted in correspondence with the positions of the pulses to illustrate the changes. The spectrum of the NO-Gd ruler is shown as a shaded gray area. (a) Echo decay experiments performed at 50 K using a shot repetition time (srt) of 1,000 µs. (b) Echo decay experiments performed on Gd at 10 K using an srt of 1,000 µs. (c) Exemplary plots of echo decay transients recorded at the NO pump position of the NO-Gd ruler sample. Shown are the used evaluation method with $T_{m\,[10\%]}$ and a stretched exponential fitting of the data with the exponent B and the resulting $T_{m\,[exp]}$. The T_m values obtained from both methods differ significantly, but the relaxation data shown in Table S4 are comparable across the samples since recorded using the same parameters and evaluated using the same method.

**sample**	**T** [K]	**$T_{m\,[10\%]}$ (NO)** [µs] **Magnetic Field Position**		**$T_{m\,[10\%]}$ (Gd)** [µs] **Magnetic Field Position**			
		NO_{pump}	NO_{obs}	NO_{pump}	NO_{obs}	Gd_{pump}	Gd_{obs}
**NO-NO**	10						
	50	4.6	4.6				
**NO-Gd**	10			4.4	4.5	4.6	4.6
	50	2.1	2.0			1.9	
**Gd-Gd**	10			4.7	4.7	5.0	4.9
	50						
**NO-NO + NO-Gd**	10			4.4	4.6	4.9	4.8
	50	3.5	3.5				
**NO-NO + 2xGd-Gd**	10			4.4	4.5	4.7	4.6
	50	4.3	4.1				
**NO-Gd + 2xGd-Gd**	10			4.6	4.7	5.0	4.9
	50	2.1	1.8				
**NO-NO + NO-Gd + 2xGd-Gd**	10			4.7	4.8	5.0	4.9
	50	3.8	3.7			**1.5**	

Table S4: Transverse $T_{m\,[10\%]}$ relaxation data obtained on all samples investigated at different temperatures. A plot of the data is presented in Fig. S2. We estimate an error of 10% based on variations when doing independent repetitions.

3.3 Optimization of the swapped NOGd DEER setup

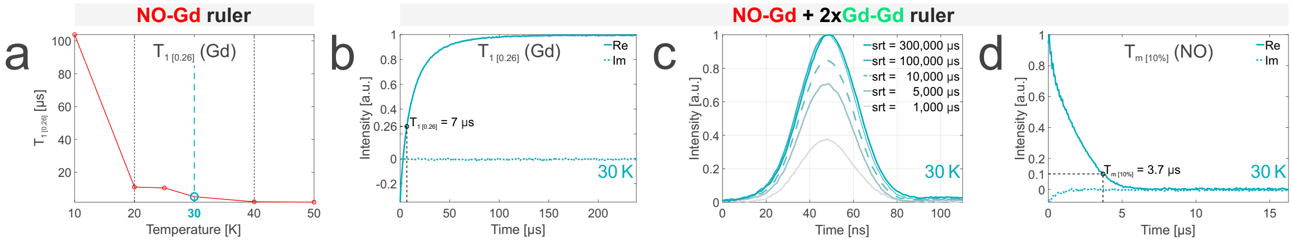

Figure S3: Temperature dependence of T_1 and $T_{m\,[10\%]}$. (a) Temperature dependence of T_1 at the maximum of the Gd spectrum measured on the isolated NO-Gd ruler sample. The highest feasible temperature for the swapped DEER setup is 30 K (highlighted in blue), where the T_1 is 5.2 µs. (b-d) NO and Gd relaxation parameters (see Fig. 3(d) and Fig. 9) at 30 K measured on the NO-Gd and Gd-Gd mixture in a 1:2 molar ratio. (b) T_1 measured at the maximum of the Gd spectrum (= pump position in the swapped NOGd DEER). (c) Transiently recorded echo on the maximum of the NO spectrum (= observer position in the swapped NOGd DEER) at different shot repetition times (srt). The srt of 10,000 µs (dashed line) was chosen to optimize the accumulation time in the swapped DEER experiments. (d) $T_{m\,[10\%]}$ measured using an srt of 10,000 µs on the maximum of the NO spectrum (= observer position in the swapped NOGd DEER).

4 DEER data

4.1 Gaussian versus Tikhonov analysis

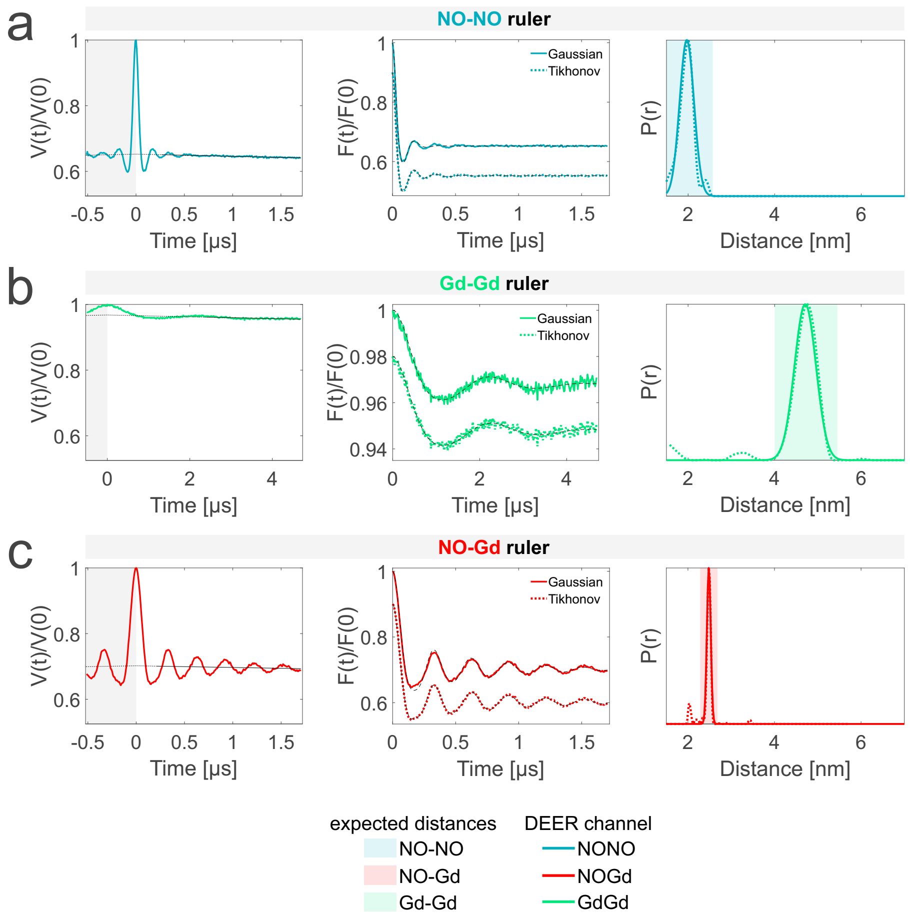

Figure S4: DEER data evaluation methods (related to Fig. 4 in the main text). Comparison of Gaussian fitting and Tikhonov regularization methods for DEER data evaluation. Left, primary data with background fit (gray areas are excluded from data evaluation); middle, form factors with fit; right, obtained distance distributions. The small distance peak at 3.5 nm in the GdGd DEER (green) is a residual artifact (see Fig. S8). The small peak at 2 nm in the NOGd DEER (red) is possibly due to residual orientation selection effects.

4.2 Quantification

Table S5 gives an overview of all DEER data presented in the main text. DEER data evaluation was performed using the Gaussian fitting routine in DeerAnalysis2019. For each sample and DEER channel, we present the temperature in K at which the experiment was performed, the length of the time trace in µs, the number of Gaussians used for the fit, the obtained modulation depth and root-mean-square deviation (rmsd) of the fit, the population of the Gaussians relative to the NO-NO (2 nm), NO-Gd (2.5 nm), Gd-Gd (4.7 nm) and artifact (3.5 nm).

Sample	DEER channel	**Temp. [K]**	Time Trace Length [µs]	Form Factor: Gaussians	Form Factor: mod. depth [%]	Form Factor: rmsd of fit	Gaussian Distance Population [%]: NO-NO	Gaussian Distance Population [%]: NO-Gd	Gaussian Distance Population [%]: Gd-Gd	Gaussian Distance Population [%]: artifact
**NO-NO**	NONO	50	1.7	1	35	0.0032	100			
	NOGd									
	GdGd									
**NO-Gd**	NONO	50	0.7	1	4	0.0070		100		
	NOGd	10	1.7	1	30	0.0049		100		
	GdGd	10	0.7		0	N/A				
**Gd-Gd**	NONO									
	NOGd									
	GdGd	10	4.7	1	3	0.0016			100	
**NO-NO + NO-Gd**	NONO	50	1.7	1	31	0.0040	100			
	NOGd	10	1.7	1	32	0.0070		100		
	GdGd	10	0.7		0	N/A				
**NO-NO + 2xGd-Gd**	NONO	50	1.7	1	37	0.0040	100			
	NOGd	10	4.7	1	4	0.0042			100	
		10	4.7	2	4	0.0032			65	35
Gd opt. pump	NOGd	10	4.7	2	1	0.0016			92	8
swapped	NOGd	30	1.7		0	N/A				
	GdGd	10	4.7	1	3	0.0019			100	
		10	4.7	2	3	0.0018			85	15
**NO-Gd + 2xGd-Gd**	NONO	50	0.7	1	2	0.0039		100		
	NOGd	10	1.7	2	14	0.0043		94	6	
		10	4.7	2	15	0.0025		91	9	
Gd opt. pump	NOGd	10	4.7	2	2	0.0008		60	40	
swapped	NOGd	30	3.0	2	5	0.0013		100	0	
	GdGd	10	4.7	1	3	0.0008			100	
**NO-NO + NO-Gd + 2xGd-Gd**	NONO	50	1.7	1	34	0.0049	100			
	NOGd	10	1.7	2	12	0.0039		92	8	
	GdGd	10	4.7	1	2	0.0018			100	

Table S5: Overview of all DEER data evaluated using the Gaussian fitting routine presented in the main text.

4.3 Equimolar ruler mixtures

The data shown in this section are independent repetions of the samples presented in the main text prepared with equimolar misxtures of the rulers. The mixtures were prepared using independently prepared stock solutions from the same batch of rulers.

4.3.1 Sample preparation

**sample**	**ruler**	$\mathbf{V_{stock}}$ [µl]	$\mathbf{c_{stock(spin)}}$ [µM]	$\mathbf{V_{d8\text{-}glycerol}}$ [µl]	$\mathbf{V_{final}}$ [µl]	$\mathbf{c_{final(spin)}}$ [µM]
**NO-NO**	NO-NO<br>NO-Gd<br>Gd-Gd	20<br>-<br>-	100<br>-<br>-	20	40	50<br>-<br>-
**NO-Gd**	NO-NO<br>NO-Gd<br>Gd-Gd	-<br>20<br>-	-<br>100<br>-	20	40	-<br>50<br>-
**Gd-Gd**	NO-NO<br>NO-Gd<br>Gd-Gd	-<br>-<br>20	-<br>-<br>100	20	40	-<br>-<br>50
**NO-NO** + **NO-Gd**	NO-NO<br>NO-Gd<br>Gd-Gd	10<br>10<br>-	100<br>100<br>-	20	40	25<br>25<br>-
**NO-NO** + **Gd-Gd**	NO-NO<br>NO-Gd<br>Gd-Gd	10<br>-<br>10	100<br>-<br>100	20	40	25<br>-<br>25
**NO-Gd** + **Gd-Gd**	NO-NO<br>NO-Gd<br>Gd-Gd	-<br>10<br>10	-<br>100<br>100	20	40	-<br>25<br>25
**NO-NO** + **NO-Gd** + **Gd-Gd**	NO-NO<br>NO-Gd<br>Gd-Gd	6.67<br>6.67<br>6.67	100<br>100<br>100	20	40	16.67<br>16.67<br>16.67

Table S6: Sample preparation of the NO-NO, NO-Gd and Gd-Gd rulers in a 1:1:1 ratio. The DEER data obtained on these samples are presented in Fig. S5 to S7.

4.3.2 DEER data

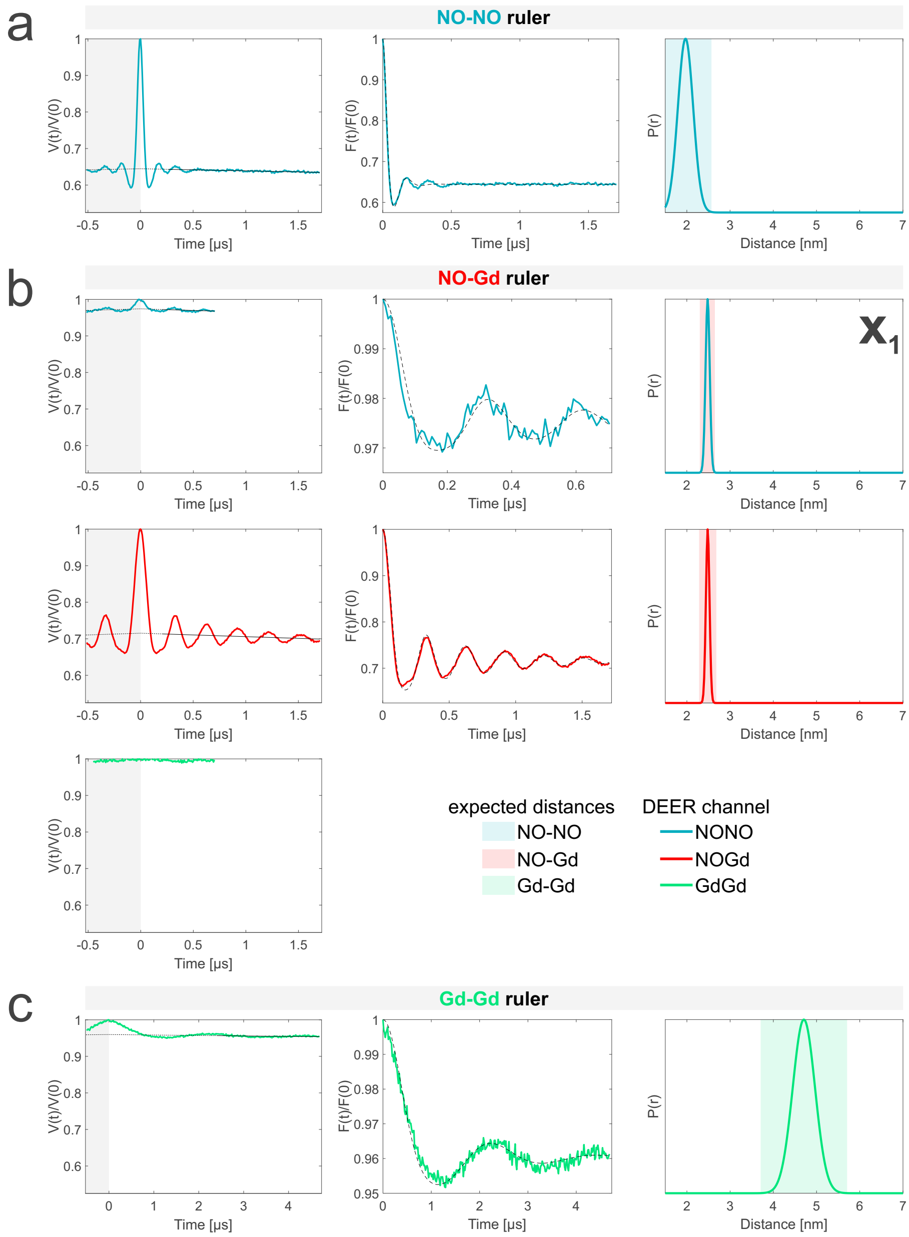

Figure S5: Characterization of the isolated rulers (repetition experiments, to be compared with those in Fig. 4). The DEER setups are introduced in Fig. 3. The time traces, form factors and distance distributions recorded with the NONO DEER channel are colored in blue, those recorded with the GdGd channel are colored in green, and those recorded with the NOGd channel are colored in red. Regions in which distances can be expected based on the rulers present in the specific sample are represented as shaded blue, green and red areas in the distance distributions. "$\mathbf{X}_1$" is a NO-Gd crosstalk in the NONO DEER channel.

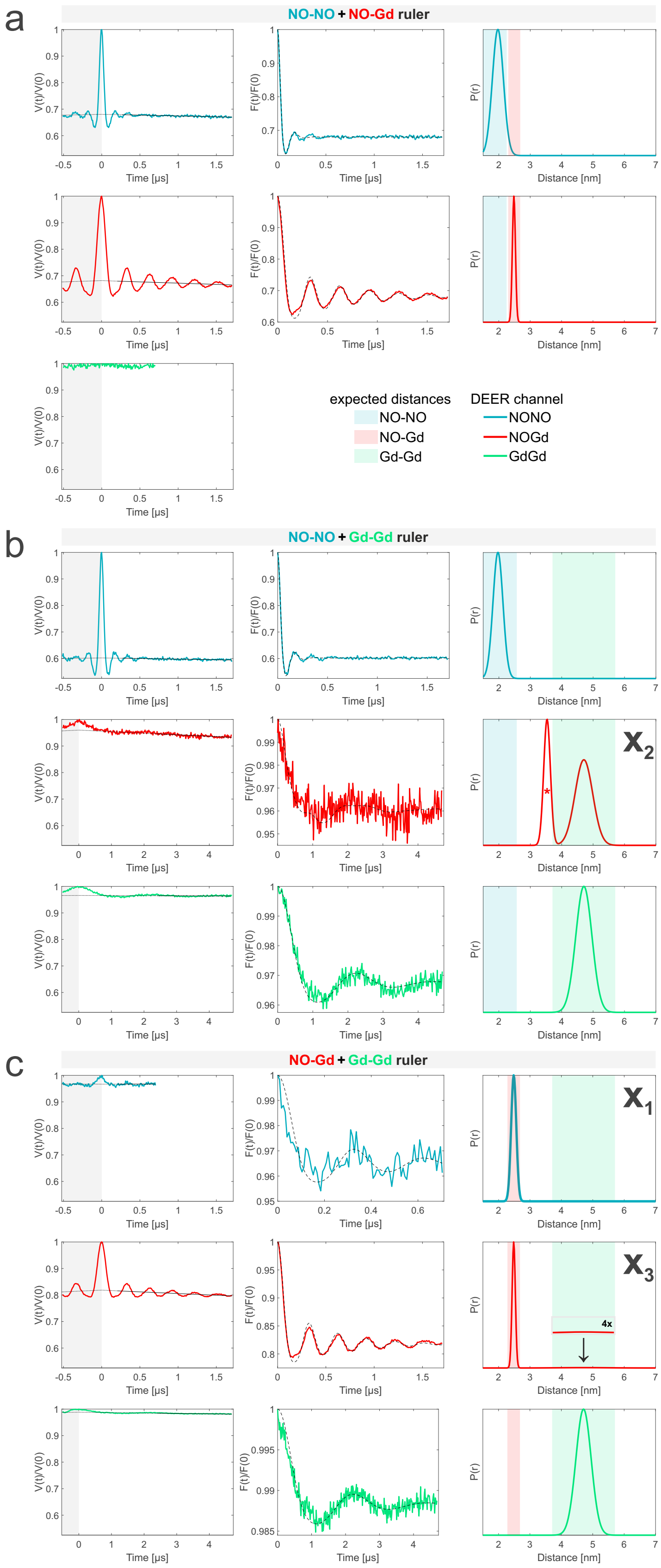

Figure S6: Samples containing pairwise mixtures of the rulers in a 1:1 molar ratio (related to Fig. 5-7 in the main text). Same figure legend as in Fig. S5. (a) Sample containing the NO-NO and the NO-Gd rulers. (b) Sample containing the NO-NO and the Gd-Gd rulers. The NOGd DEER channel contains a Gd-Gd crosstalk signal in absence of a NO-Gd distance designated as $\mathbf{X}_2$. The distance indicated with an asterisk originates from the artifact signal. (c) Sample containing the NO-Gd and the Gd-Gd rulers. The NONO DEER channel contains a NO-Gd crosstalk signal ($\mathbf{X}_1$). Notably, the $\mathbf{X}_3$ crosstalk signal detected on the 1:2 mixture in Fig. 7 is negligible in this sample with equimolar ratios of rulers.

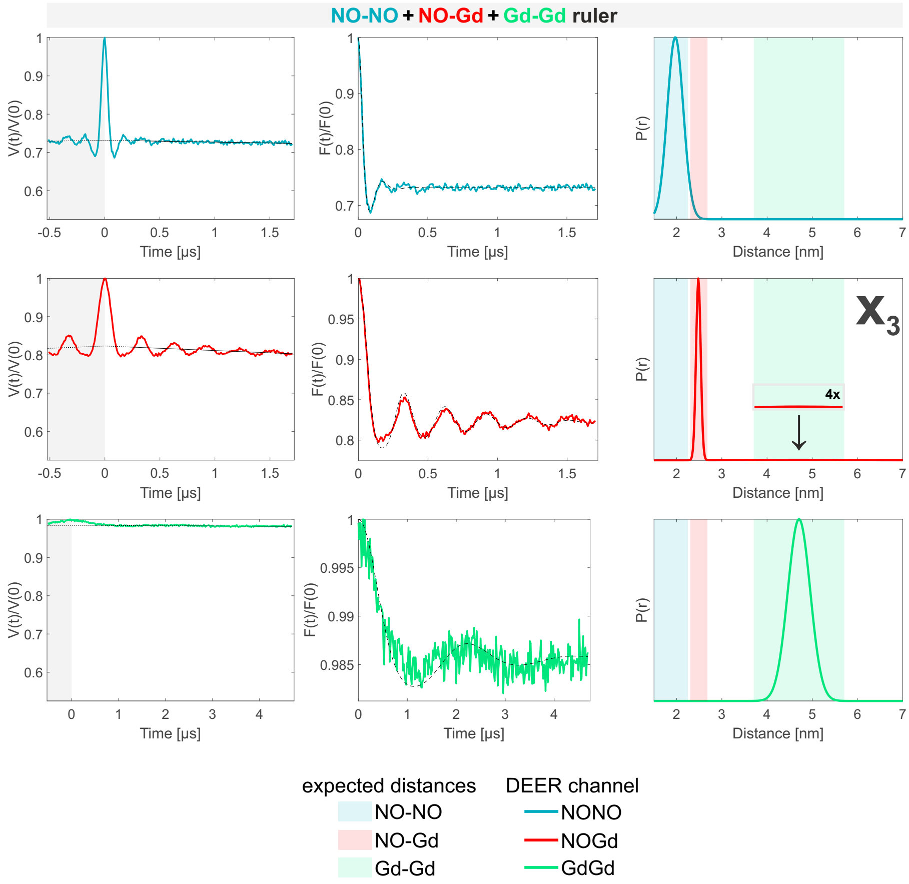

Figure S7: Sample containing the NO-NO, NO-Gd and Gd-Gd rulers mixed in a 1:1:1 ratio. (related to Fig. 8 in the main text). The legend is the same as in Fig. S5. Notably, the $\mathbf{X}_3$ crosstalk signal detected on the 1:1:2 mixture in Fig 8 is negligible in this sample with equimolar ratios of rulers.

4.4 Spectrometer artifact

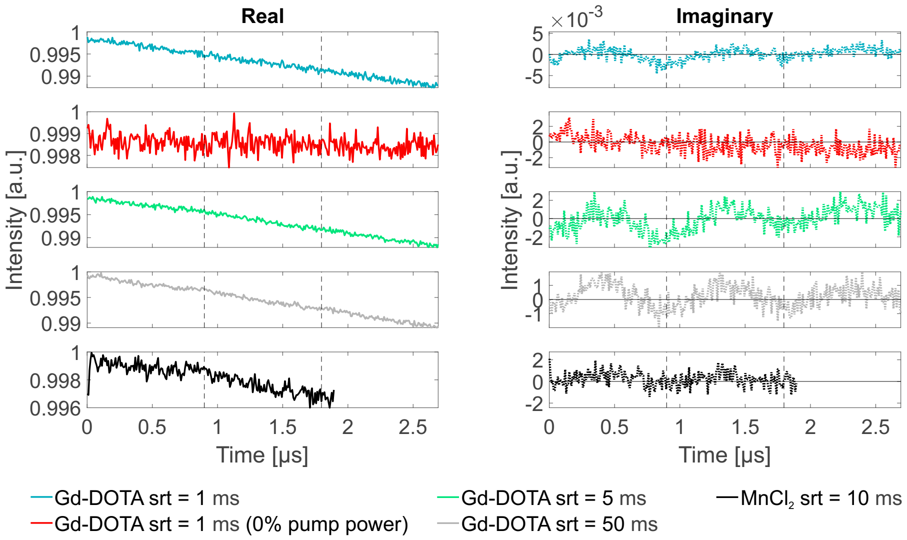

Figure S8: Characterization of the spectrometer artifact. DEER experiments were performed on solutions of 40 μ maleimide Gd-DOTA and 50 μM $MnCl_2$ in water with deuterated glycerol. The length of the pulses were optimized by nutation experiments if not otherwise stated. Left, real part; right, imaginary part of the DEER data starting from the zero dipolar evolution time. Traces are obtained with different srt, written in the legend, and in one case the power of the pump pulse was set to zero. The artifact is mostly visible in the Imaginary part of the signal, it is a sinusoidal oscillation with a period of 0.9 μs, which is residually present in some DEER time traces (e.g. in the NOGd and GdGd channel in Fig. 6). The artifact has no dipolar origin, being visible in the isolated Gd labels, it is independent on the spin system and has no hyperfine origin (being visible also in the Mn system). It can be strongly reduced if the pump pulse amplitude is set to zero (red trace). We could not find the origin of such artifact, therefore, we speculate that it is a spectrometer- or AWG-related oscillation.

4.5 DeerNet

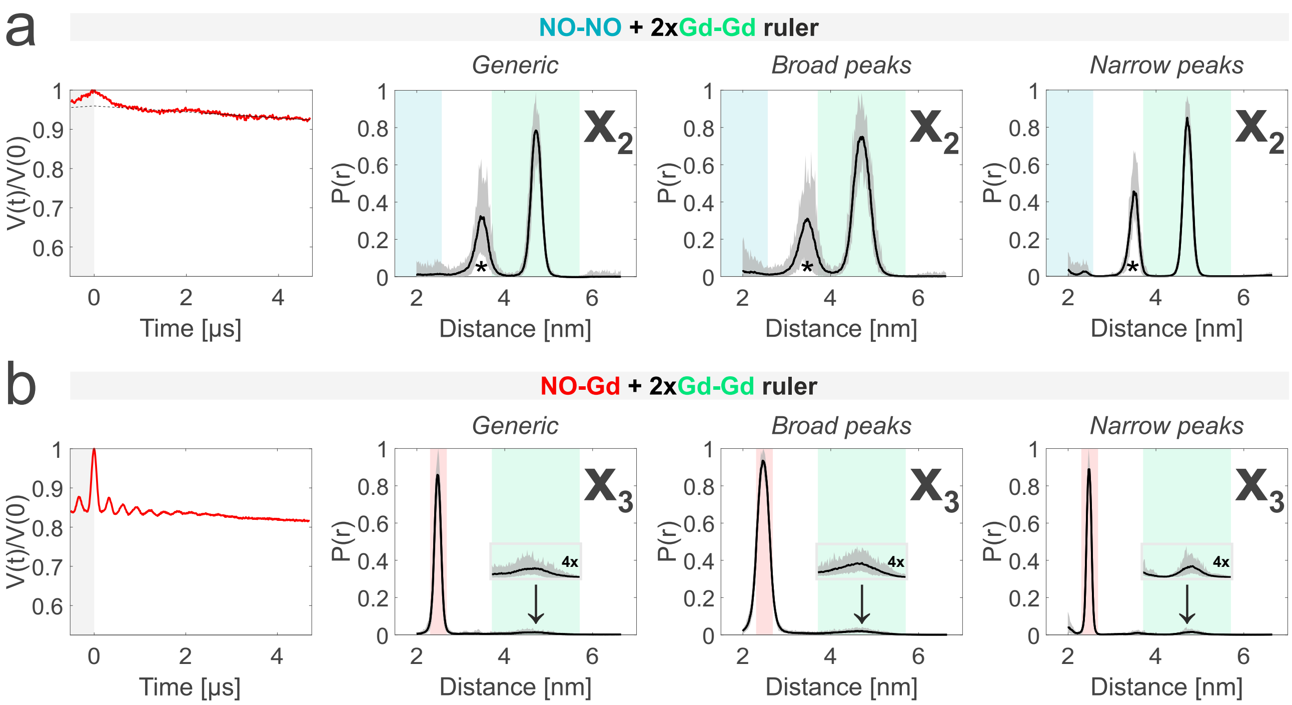

Figure S9: Comparison of different DeerNets trained to fit specific types of distances (related to Fig. 6 and 7 in the main text). First column, primary data; second to fourth column, DeerNet distance distributions. In the main figures, only the Generic network is shown since this should provide the best overall performance and does not require any previous knowledge about the system. Here, we compare the outputs of networks specifically optimized to fit broad and narrow distance peaks. (a) Sample containing the NO-NO and the Gd-Gd rulers mixed in a 1:2 molar ratio (related to the NOGd DEER channel in Fig. 6). All DeerNets fit the crosstalk signal with high significance. The intensity of the spectrometer artifact signal (asterisk) depends on the chosen network. (b) Sample containing the NO-Gd and the Gd-Gd rulers mixed in a 1:2 molar ratio (related to the NOGd DEER channel in Fig. 7). All networks show a significant crosstalk distance peak at the Gd-Gd distance position.

4.6 Crosstalk signal overview

sample	experiment	NO-NO signal poss.	NO-NO signal exp.	NO-Gd signal poss.	NO-Gd signal exp.	Gd-Gd signal poss.	Gd-Gd signal exp.
**NO-NO**	NONO DEER	+	+				
	NOGd DEER						
	GdGd DEER						
**NO-Gd**	NONO DEER			$\mathbf{X}_1$	$\mathbf{X}_1$		
	NOGd DEER			+	+		
	GdGd DEER						
**Gd-Gd**	NONO DEER						
	NOGd DEER						
	GdGd DEER					+	+
**NO-NO + NO-Gd**	NONO DEER	+	+	$\mathbf{X}_1$			
	NOGd DEER			+	+		
	GdGd DEER						
**NO-NO + 2xGd-Gd**	NONO DEER	+	+			$\mathbf{X}_4$	
	NOGd DEER					$\mathbf{X}_2$	$\mathbf{X}_2$
	GdGd DEER					+	+
**NO-Gd + 2xGd-Gd**	NONO DEER			$\mathbf{X}_1$	$\mathbf{X}_1$	$\mathbf{X}_4$	
	NOGd DEER			+	+	$\mathbf{X}_3$	$\mathbf{X}_3$
	GdGd DEER					+	+
**NO-NO + NO-Gd + 2xGd-Gd**	NONO DEER	+	+	$\mathbf{X}_1$		$\mathbf{X}_4$	
	NOGd DEER			+	+	$\mathbf{X}_3$	$\mathbf{X}_3$
	GdGd DEER					+	+

Table S7: This table summarizes crosstalk signals between NO and Gd that are possible based on the spectral overlap in the observer and/or pump position ("poss.") versus those that were actually experimentally detected ("exp.") in all samples using the conventional DEER setups given in Fig. 3a-c. The white cells left represent signals which cannot be expected in the setup/samples used. The other cells are colored as follows: desired dipolar frequency + / crosstalk $\mathbf{X}_i$ / not detected. The crosstalk signals are: $\mathbf{X}_1$ is an NO-Gd crosstalk signal in the NONO channel; $\mathbf{X}_2$ ($\mathbf{X}_3$) is a Gd-Gd crosstalk signal in the NOGd channel in absence (presence) of NO-Gd distance; $\mathbf{X}_4$ is a Gd-Gd crosstalk signal in the NONO channel. The table shows data obtained on the 1:1:2 mixtures, which were shown to promote the Gd-Gd crosstalk signals.

4.7 Crosstalk identification and suppression

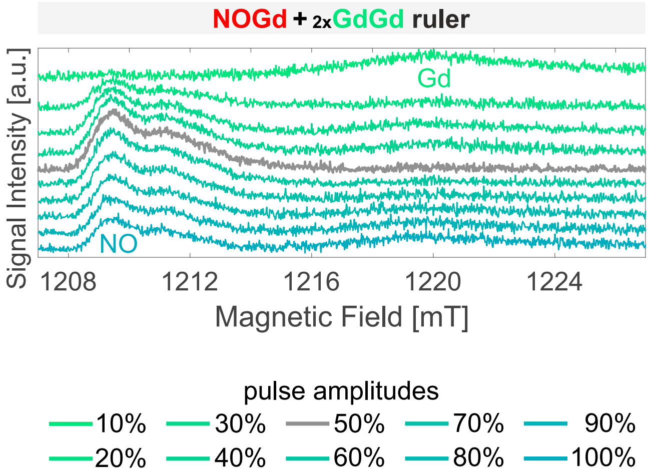

Figure S10: Additional information for the crosstalk signal suppression. The absolute values of field-swept echo (FSE)-detected spectra detected using a refocused Hahn echo sequence (DEER observer sequence) on the 1:2 mixture of the NO-Gd and Gd-Gd ruler are shown. The experiments were performed at 10 K with a shot repetition time of 100 ms. The pulses are placed at a frequency where 100% pulse amplitude corresponds to a π-pulse on the NO. Due to the distinct transition moments of NO and Gd, the relative intensities of the spectral contributions change when varying the pulse amplitudes. At 50% amplitude only the NO spectrum is refocused. This could offer an additional possibility to clean the observer echo in the swapped DEER setup shown in Fig. 3d.

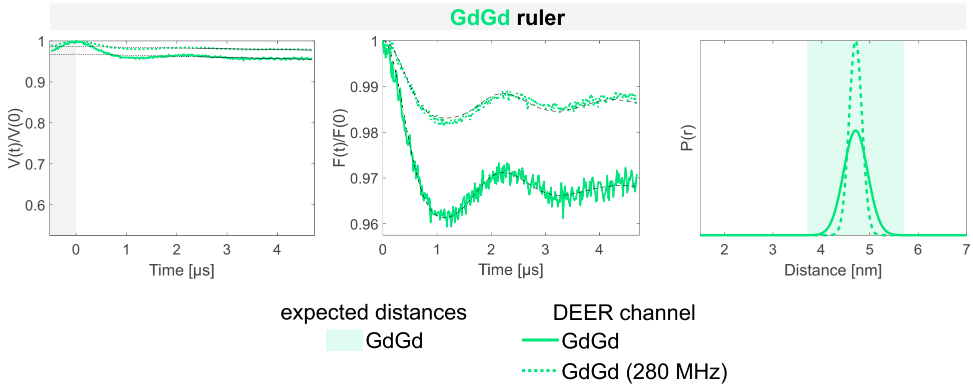

Figure S11: Comparison of GdGd DEER setups (related to Fig. 7 in the main text). Left, primary data with background fit (gray area is excluded from data evaluation); middle, form factors with Gaussian fit; right, obtained distance distributions. The data plotted as a green solid line were obtained using the standard GdGd DEER setup shown in Fig. 3(c) with the pump pulse placed on the maximum of the Gd spectrum and the observer 100 MHz lower in frequency. The second setup (green dotted line) is basically the same as the NOGd DEER setup shown in Fig. 3(b) with the observer on the spectral maximum and the pump placed 280 MHz higher in frequency but in this case with a pump pulse optimized in power to optimally excite the Gd. The data obtained with the standard GdGd DEER setup are characterized by a 3.5% modulation depth while the second setup shows a 1.25% modulation depth due to the lower spectral density at the pump position.
